# Supplementary figures and images for: REG γ knockdown suppresses proliferation by inducing apoptosis and cell cycle arrest in osteosarcoma
Source: PeerJ. 2020 Apr 15;8:e8954. doi: 10.7717/peerj.8954 (PMC7166046; doi:10.7717/peerj.8954)

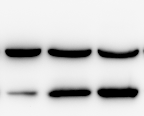

Supplement: Supplemental Information 1 [file peerj-08-8954-s001.zip › Figure 1 raw data/nomal cell line and two osteosarcoma cell lines.png]

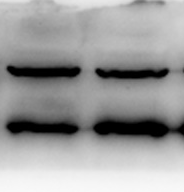

Supplement: Supplemental Information 1 [file peerj-08-8954-s001.zip › Figure 1 raw data/tissue 1 actin and REGγ.png]

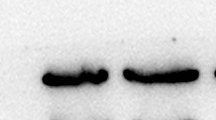

Supplement: Supplemental Information 1 [file peerj-08-8954-s001.zip › Figure 1 raw data/tissue 10 actin.png]

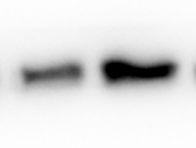

Supplement: Supplemental Information 1 [file peerj-08-8954-s001.zip › Figure 1 raw data/tissue 10 REGγ.png]

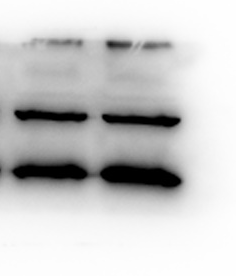

Supplement: Supplemental Information 1 [file peerj-08-8954-s001.zip › Figure 1 raw data/tissue 2 actin and REGγ.png]

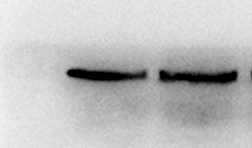

Supplement: Supplemental Information 1 [file peerj-08-8954-s001.zip › Figure 1 raw data/tissue 3 actin.png]

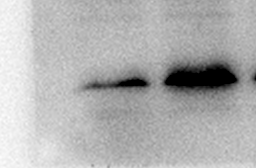

Supplement: Supplemental Information 1 [file peerj-08-8954-s001.zip › Figure 1 raw data/tissue 3 REGγ.png]

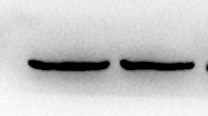

Supplement: Supplemental Information 1 [file peerj-08-8954-s001.zip › Figure 1 raw data/tissue 4 actin.png]

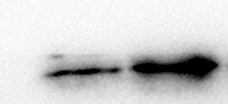

Supplement: Supplemental Information 1 [file peerj-08-8954-s001.zip › Figure 1 raw data/tissue 4 REGγ.png]

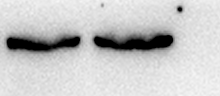

Supplement: Supplemental Information 1 [file peerj-08-8954-s001.zip › Figure 1 raw data/tissue 5 actin.png]

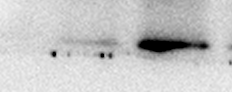

Supplement: Supplemental Information 1 [file peerj-08-8954-s001.zip › Figure 1 raw data/tissue 5 REGγ.png]

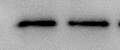

Supplement: Supplemental Information 1 [file peerj-08-8954-s001.zip › Figure 1 raw data/tissue 6 actin.png]

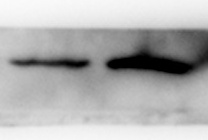

Supplement: Supplemental Information 1 [file peerj-08-8954-s001.zip › Figure 1 raw data/tissue 6 REGγ.png]

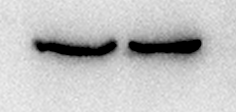

Supplement: Supplemental Information 1 [file peerj-08-8954-s001.zip › Figure 1 raw data/tissue 7 actin.png]

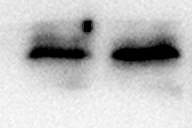

Supplement: Supplemental Information 1 [file peerj-08-8954-s001.zip › Figure 1 raw data/tissue 7 REGγ.png]

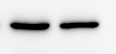

Supplement: Supplemental Information 1 [file peerj-08-8954-s001.zip › Figure 1 raw data/tissue 8 actin.png]

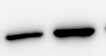

Supplement: Supplemental Information 1 [file peerj-08-8954-s001.zip › Figure 1 raw data/tissue 8 REGγ.png]

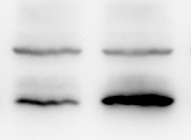

Supplement: Supplemental Information 1 [file peerj-08-8954-s001.zip › Figure 1 raw data/tissue 9 actin and REGγ.png]

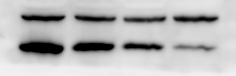

Supplement: Supplemental Information 3 [file peerj-08-8954-s002.zip › Figure 2 raw data/Mg-63 knockdown confirmation.png]

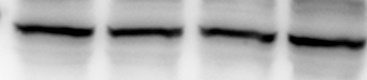

Supplement: Supplemental Information 3 [file peerj-08-8954-s002.zip › Figure 2 raw data/Saos-2 knockdown confirmation-actin.png]

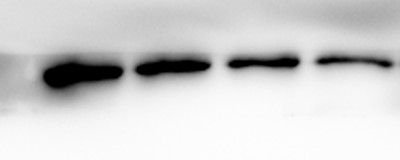

Supplement: Supplemental Information 3 [file peerj-08-8954-s002.zip › Figure 2 raw data/Saos-2 knockdown confirmation-REGγ.png]

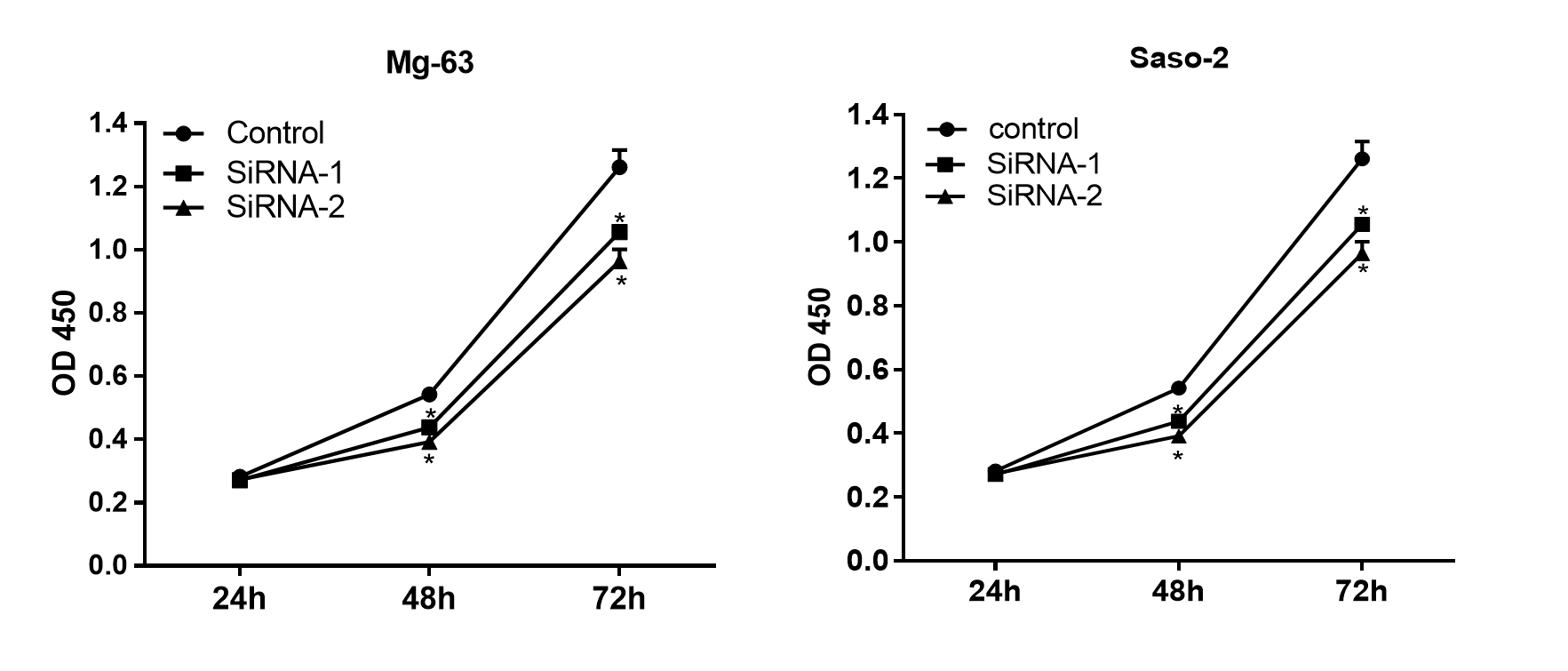

Supplement: Supplemental Information 3 [file peerj-08-8954-s003.zip › FIgure 3 raw data-1/CCK-8.png]

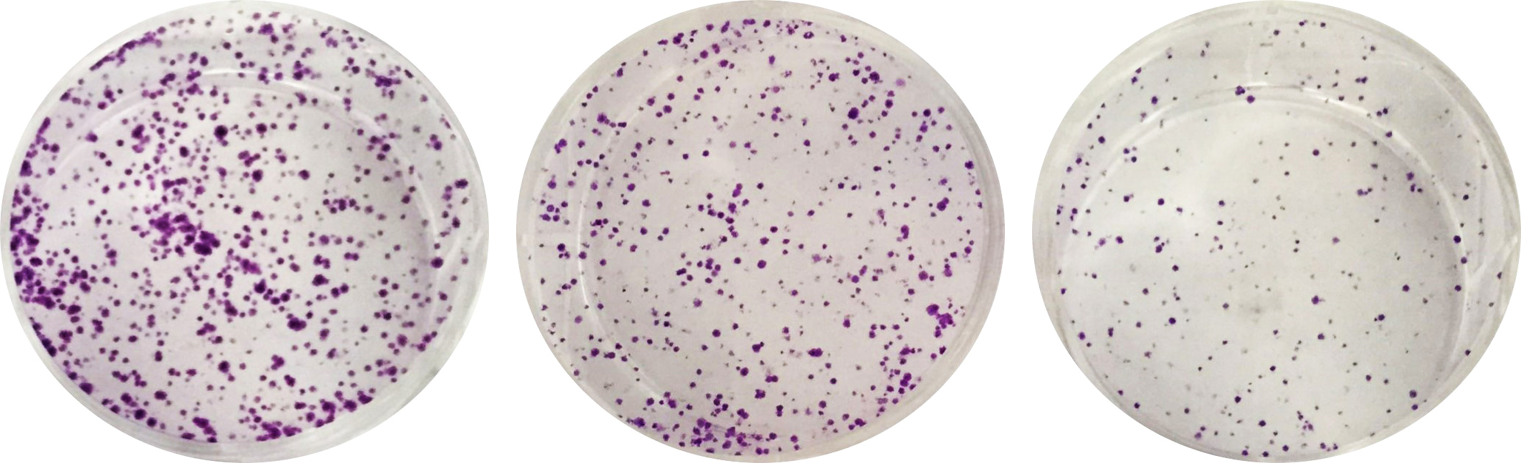

Supplement: Supplemental Information 3 [file peerj-08-8954-s003.zip › FIgure 3 raw data-1/clone formation/m.png]

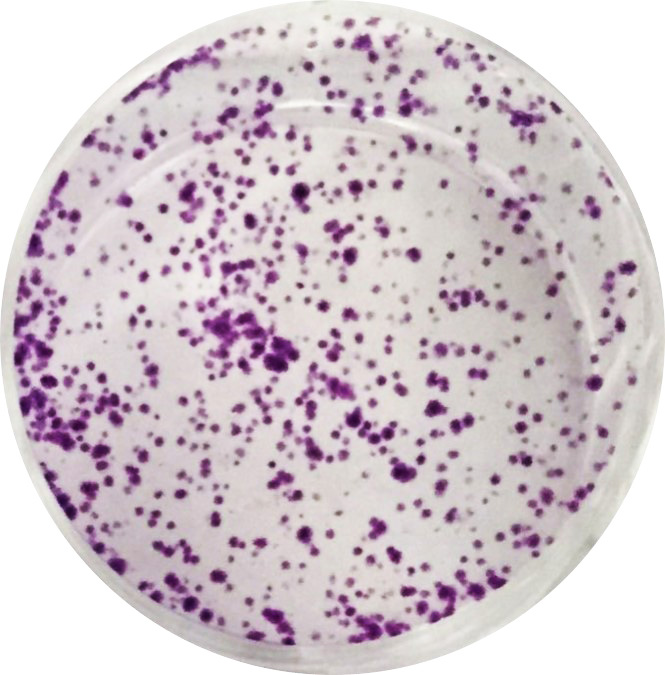

Supplement: Supplemental Information 3 [file peerj-08-8954-s003.zip › FIgure 3 raw data-1/clone formation/MG-63-siNC (2).jpg]

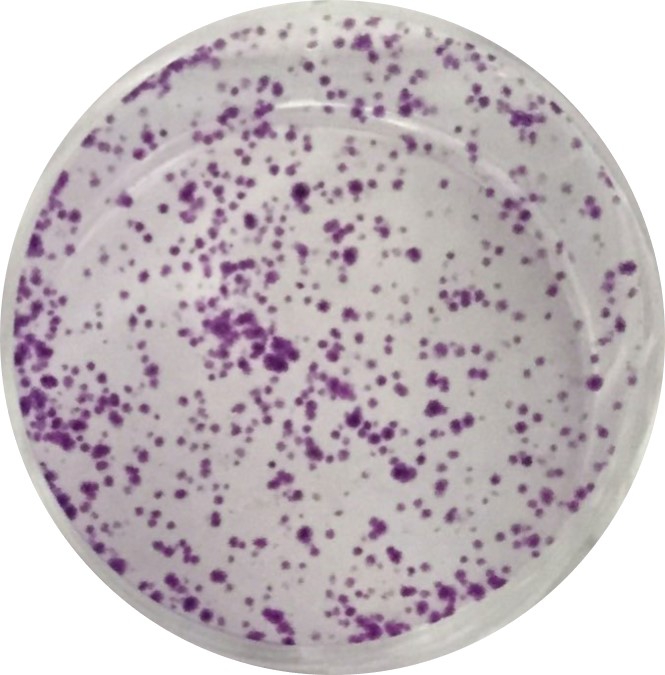

Supplement: Supplemental Information 3 [file peerj-08-8954-s003.zip › FIgure 3 raw data-1/clone formation/MG-63-siNC.jpg]

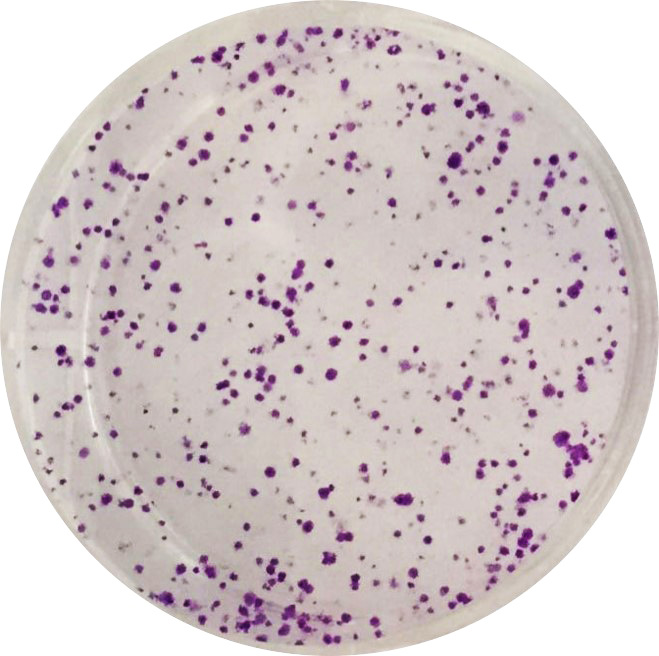

Supplement: Supplemental Information 3 [file peerj-08-8954-s003.zip › FIgure 3 raw data-1/clone formation/MG-63-siRNA-1 (2).jpg]

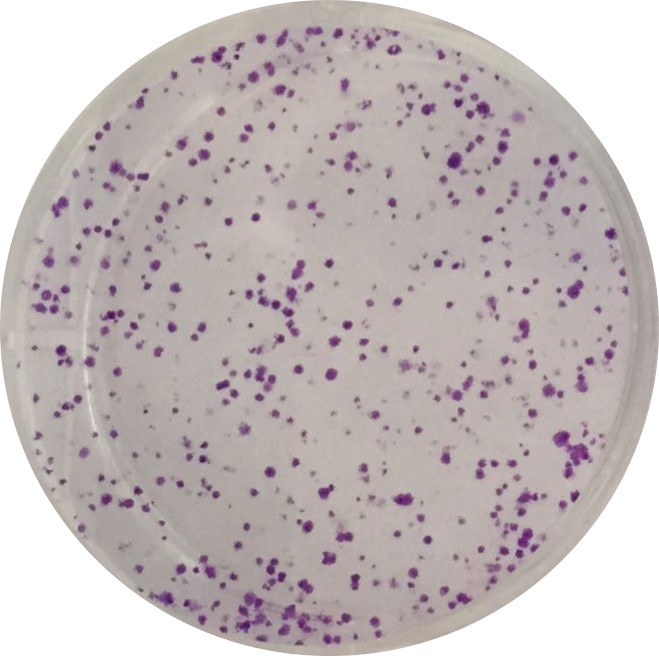

Supplement: Supplemental Information 3 [file peerj-08-8954-s003.zip › FIgure 3 raw data-1/clone formation/MG-63-siRNA-1.jpg]

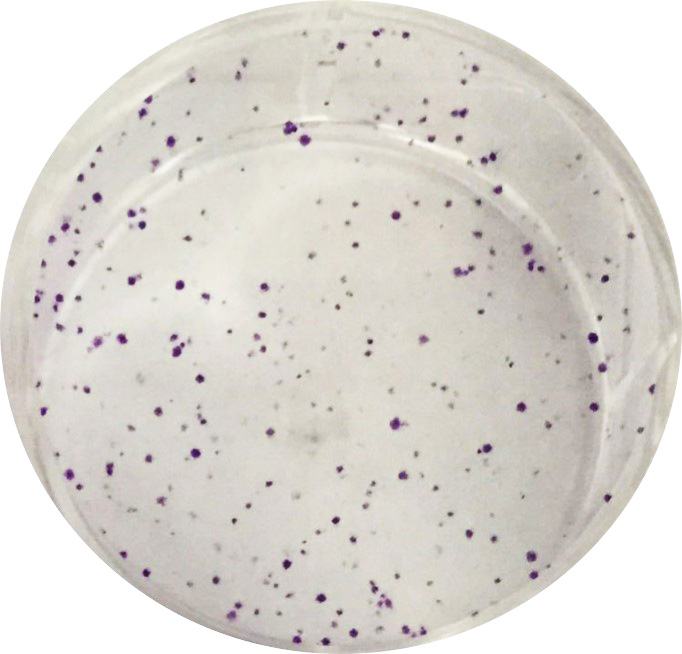

Supplement: Supplemental Information 3 [file peerj-08-8954-s003.zip › FIgure 3 raw data-1/clone formation/MG-63-siRNA-2 (2).jpg]

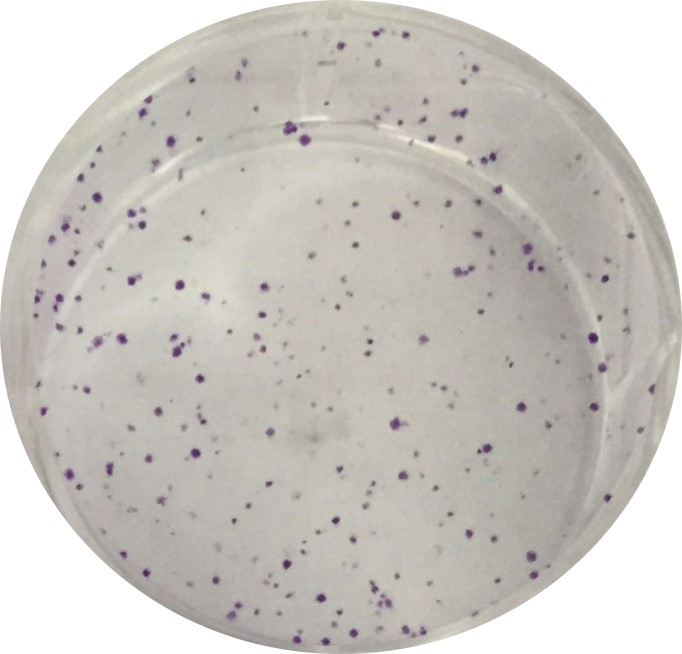

Supplement: Supplemental Information 3 [file peerj-08-8954-s003.zip › FIgure 3 raw data-1/clone formation/MG-63-siRNA-2.jpg]

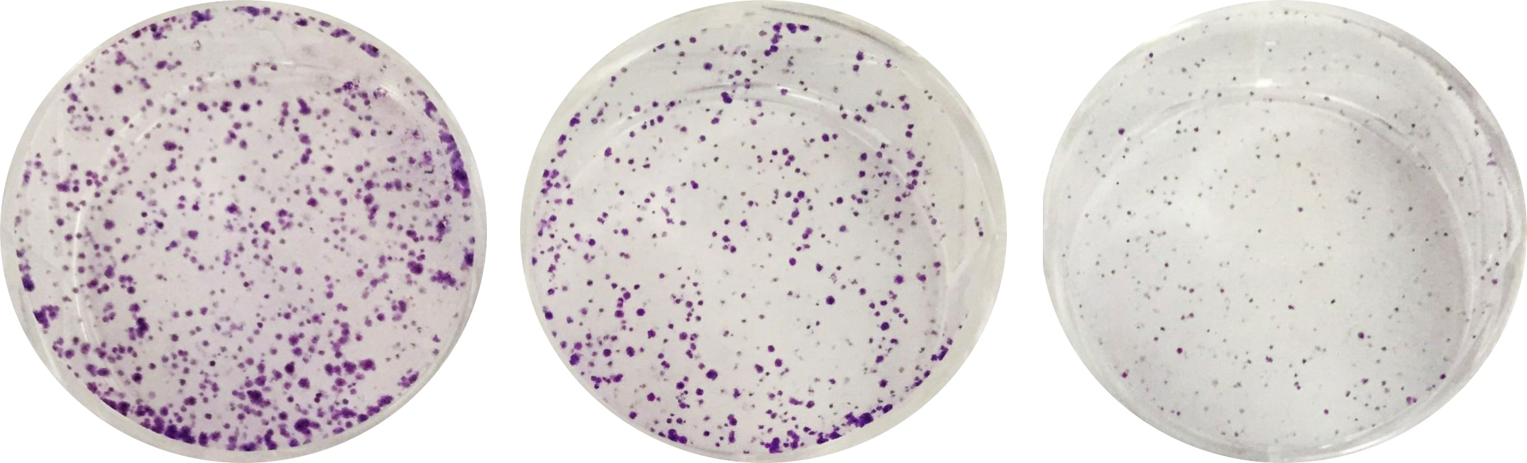

Supplement: Supplemental Information 3 [file peerj-08-8954-s003.zip › FIgure 3 raw data-1/clone formation/s.png]

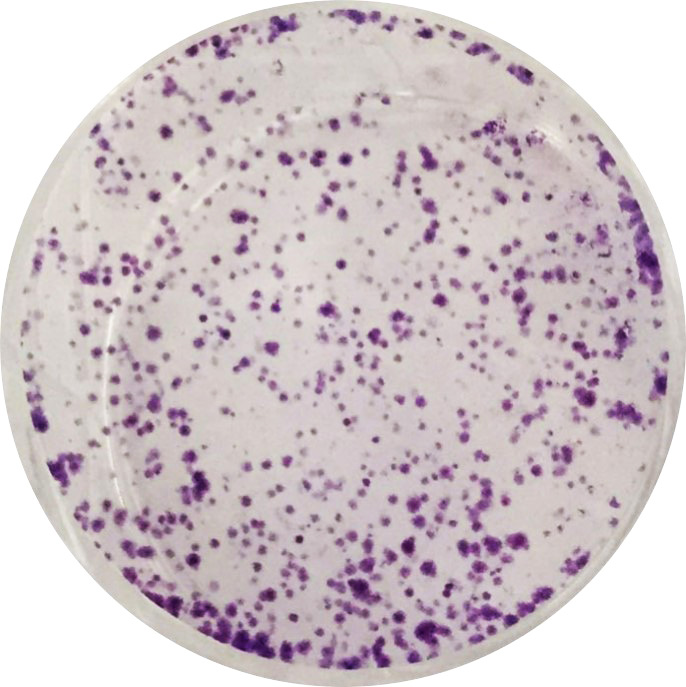

Supplement: Supplemental Information 3 [file peerj-08-8954-s003.zip › FIgure 3 raw data-1/clone formation/Saos-2-siNC (2).jpg]

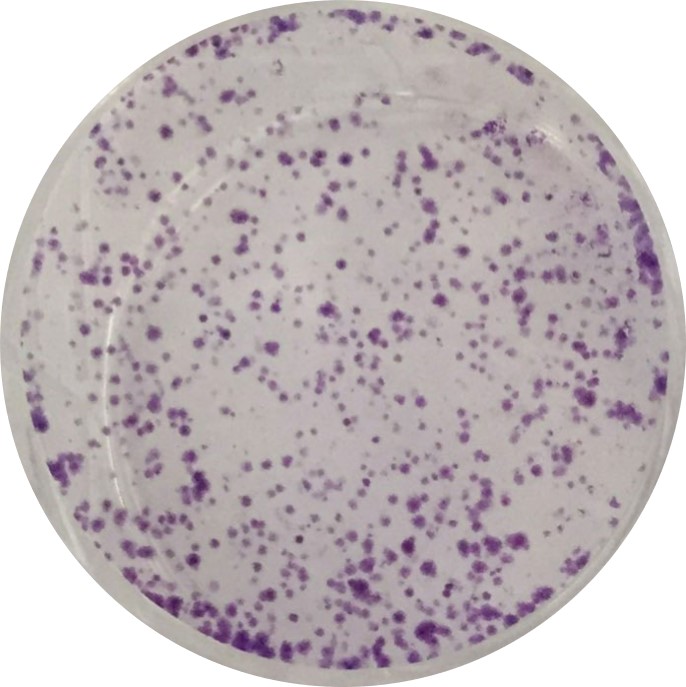

Supplement: Supplemental Information 3 [file peerj-08-8954-s003.zip › FIgure 3 raw data-1/clone formation/Saos-2-siNC.jpg]

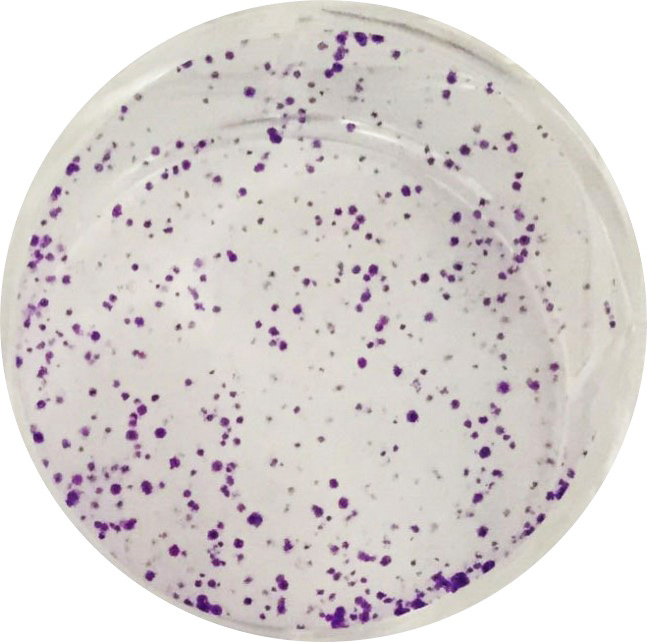

Supplement: Supplemental Information 3 [file peerj-08-8954-s003.zip › FIgure 3 raw data-1/clone formation/Saos-2-siRNA-1 (2).jpg]

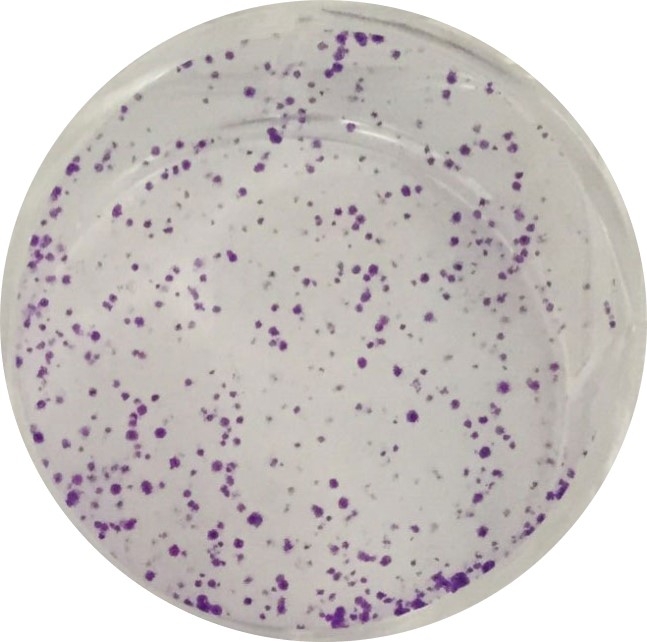

Supplement: Supplemental Information 3 [file peerj-08-8954-s003.zip › FIgure 3 raw data-1/clone formation/Saos-2-siRNA-1.jpg]

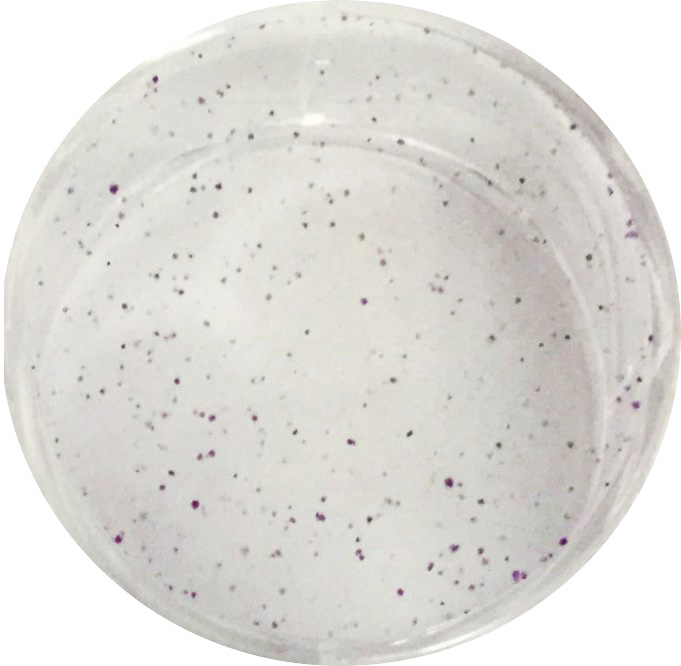

Supplement: Supplemental Information 3 [file peerj-08-8954-s003.zip › FIgure 3 raw data-1/clone formation/Saos-2-siRNA-2 (2).jpg]

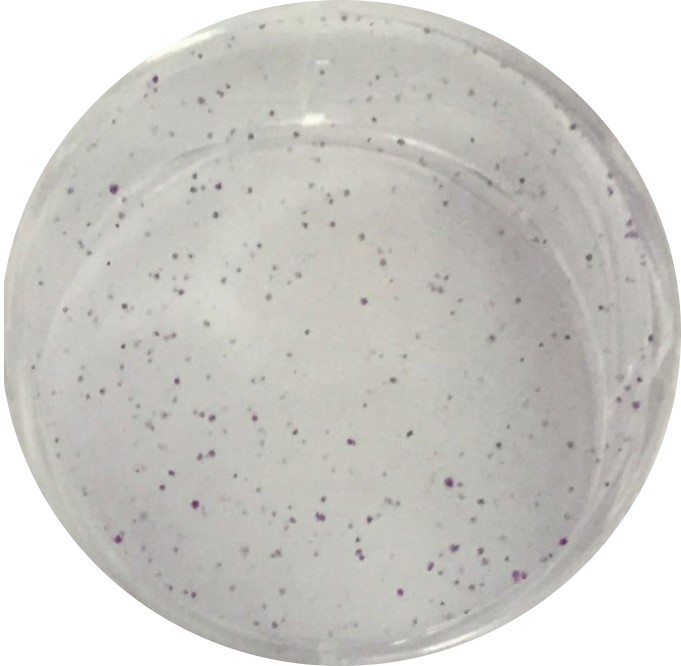

Supplement: Supplemental Information 3 [file peerj-08-8954-s003.zip › FIgure 3 raw data-1/clone formation/Saos-2-siRNA-2.jpg]

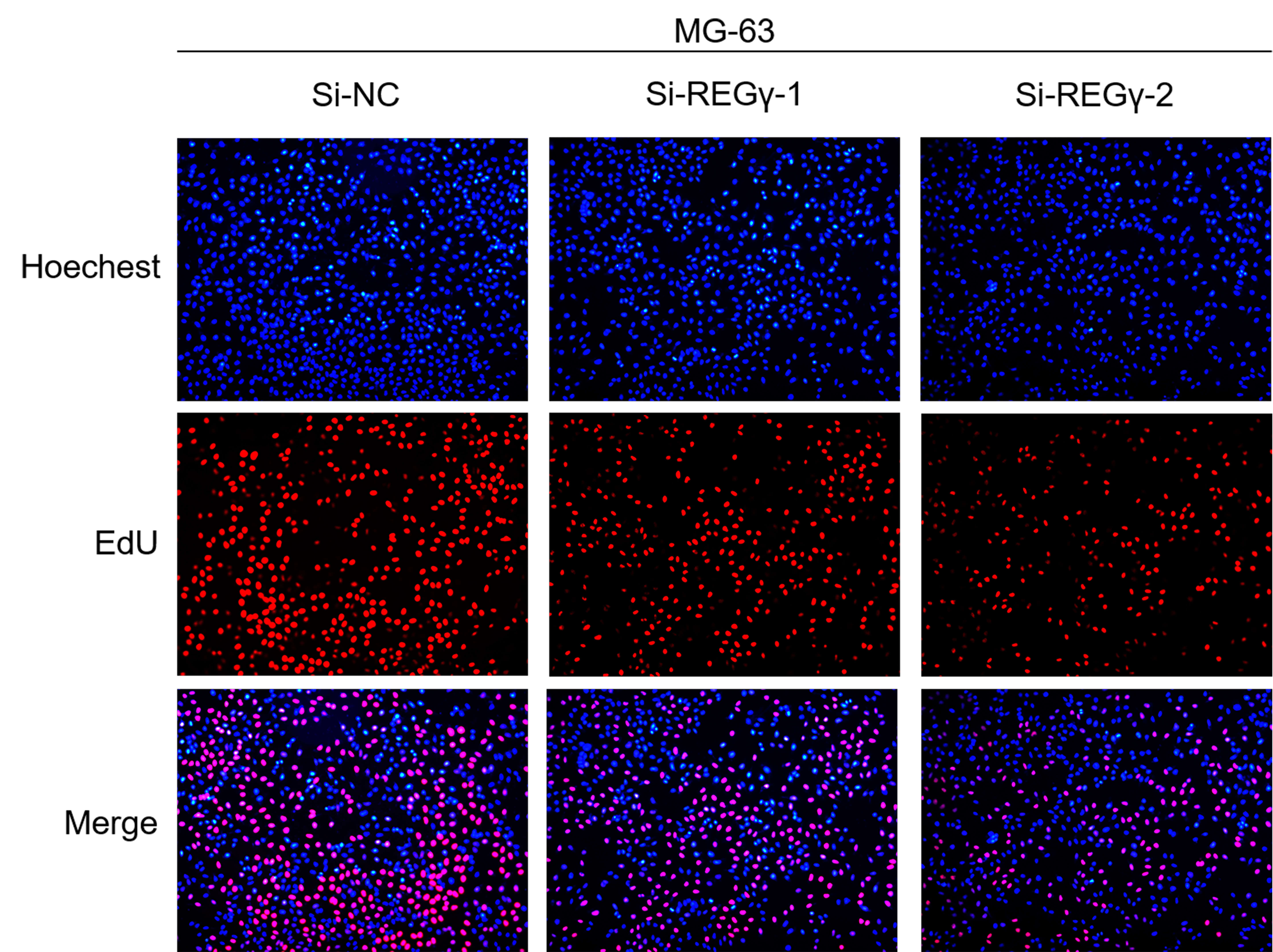

Supplement: Supplemental Information 4 [file peerj-08-8954-s004.zip › Figure 3 raw data-2/EDU-MG-63.tif]

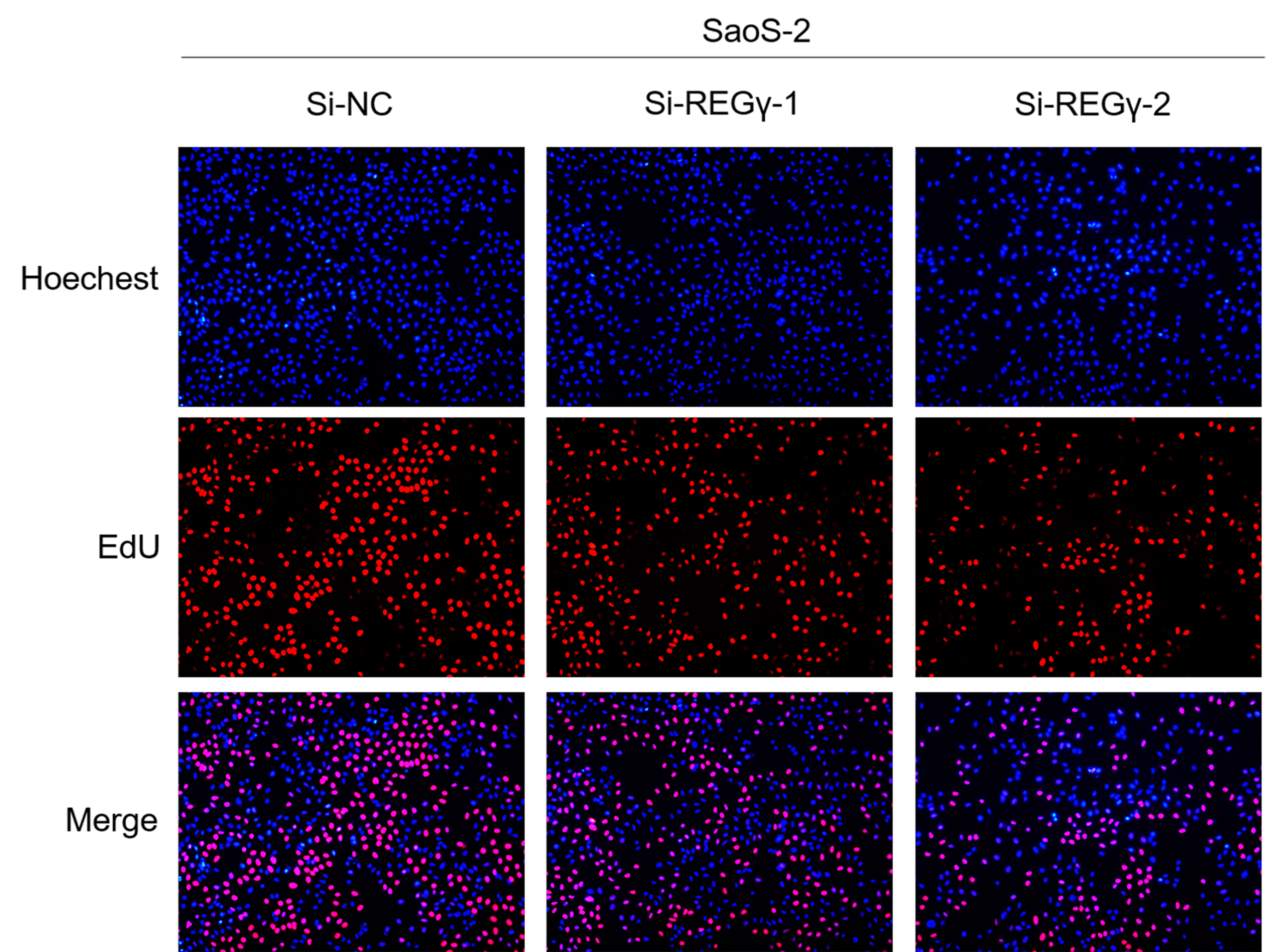

Supplement: Supplemental Information 5 [file peerj-08-8954-s005.zip › Figure 3 raw data-3/EDU-SaoS-2.tif]

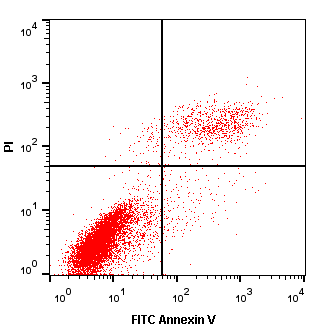

Supplement: Supplemental Information 6 [file peerj-08-8954-s006.zip › Figure 4 raw data-1/FCM-apoptosis/mg-63/1-Layout.png]

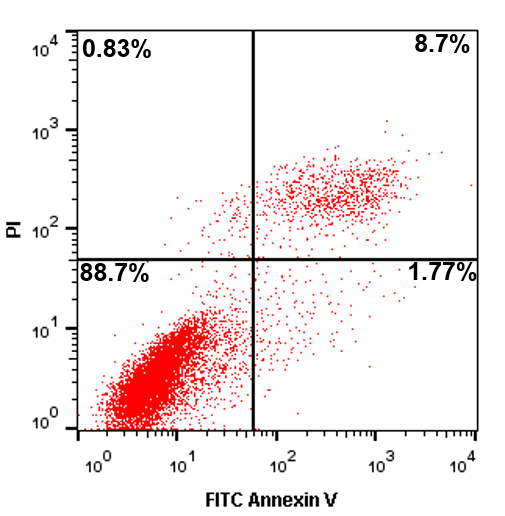

Supplement: Supplemental Information 6 [file peerj-08-8954-s006.zip › Figure 4 raw data-1/FCM-apoptosis/mg-63/1.png]

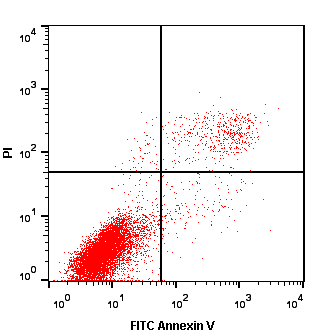

Supplement: Supplemental Information 6 [file peerj-08-8954-s006.zip › Figure 4 raw data-1/FCM-apoptosis/mg-63/2-Layout.png]

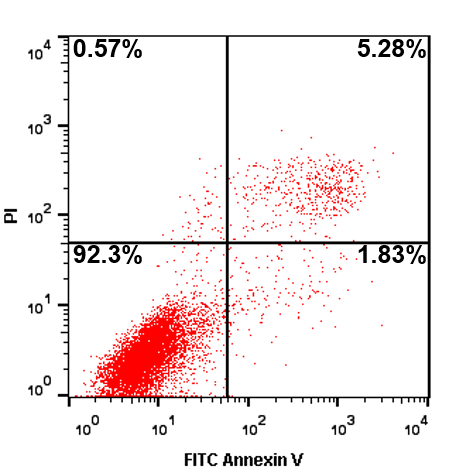

Supplement: Supplemental Information 6 [file peerj-08-8954-s006.zip › Figure 4 raw data-1/FCM-apoptosis/mg-63/2.png]

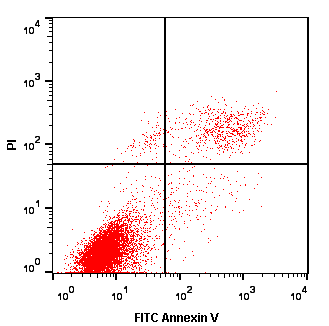

Supplement: Supplemental Information 6 [file peerj-08-8954-s006.zip › Figure 4 raw data-1/FCM-apoptosis/mg-63/3-Layout.png]

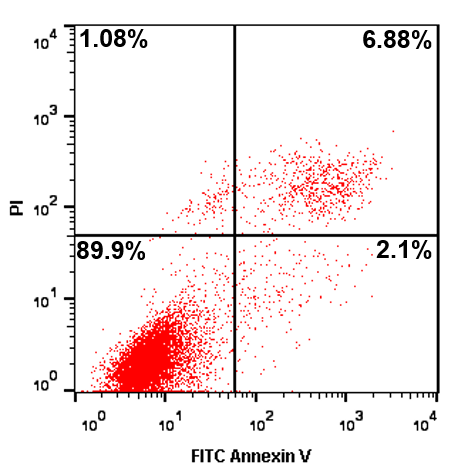

Supplement: Supplemental Information 6 [file peerj-08-8954-s006.zip › Figure 4 raw data-1/FCM-apoptosis/mg-63/3.png]

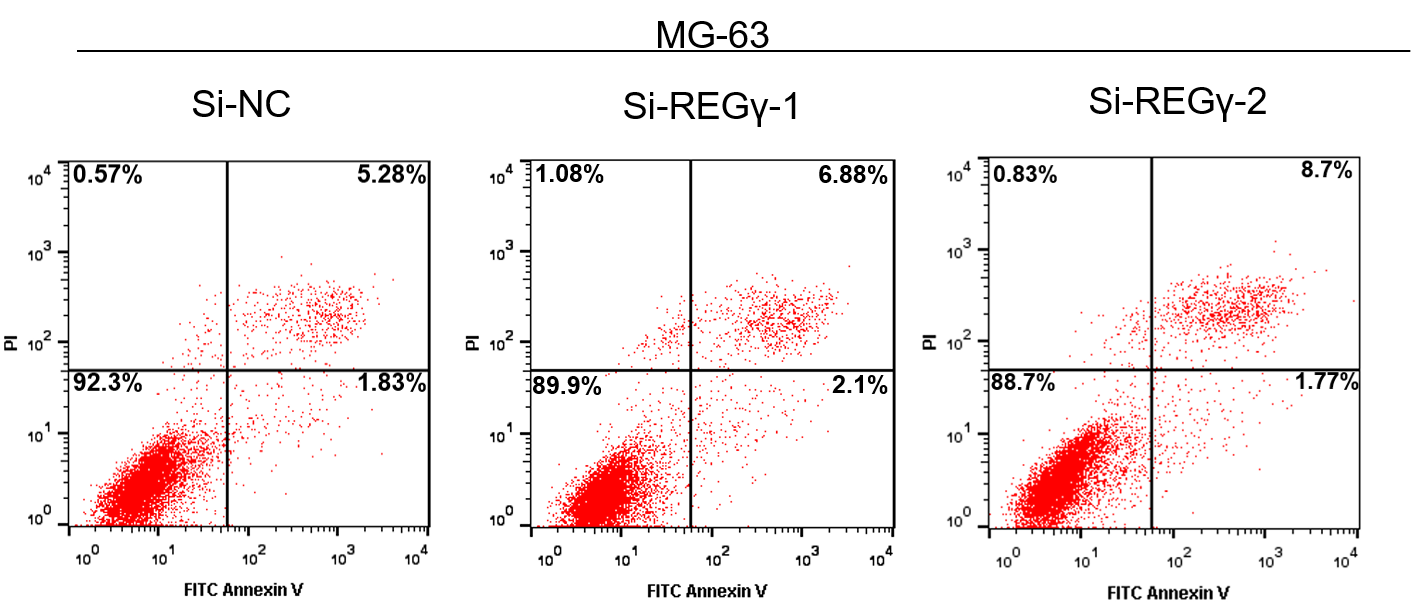

Supplement: Supplemental Information 6 [file peerj-08-8954-s006.zip › Figure 4 raw data-1/FCM-apoptosis/mg-63/Mg-63 apotosis zuhe.png]

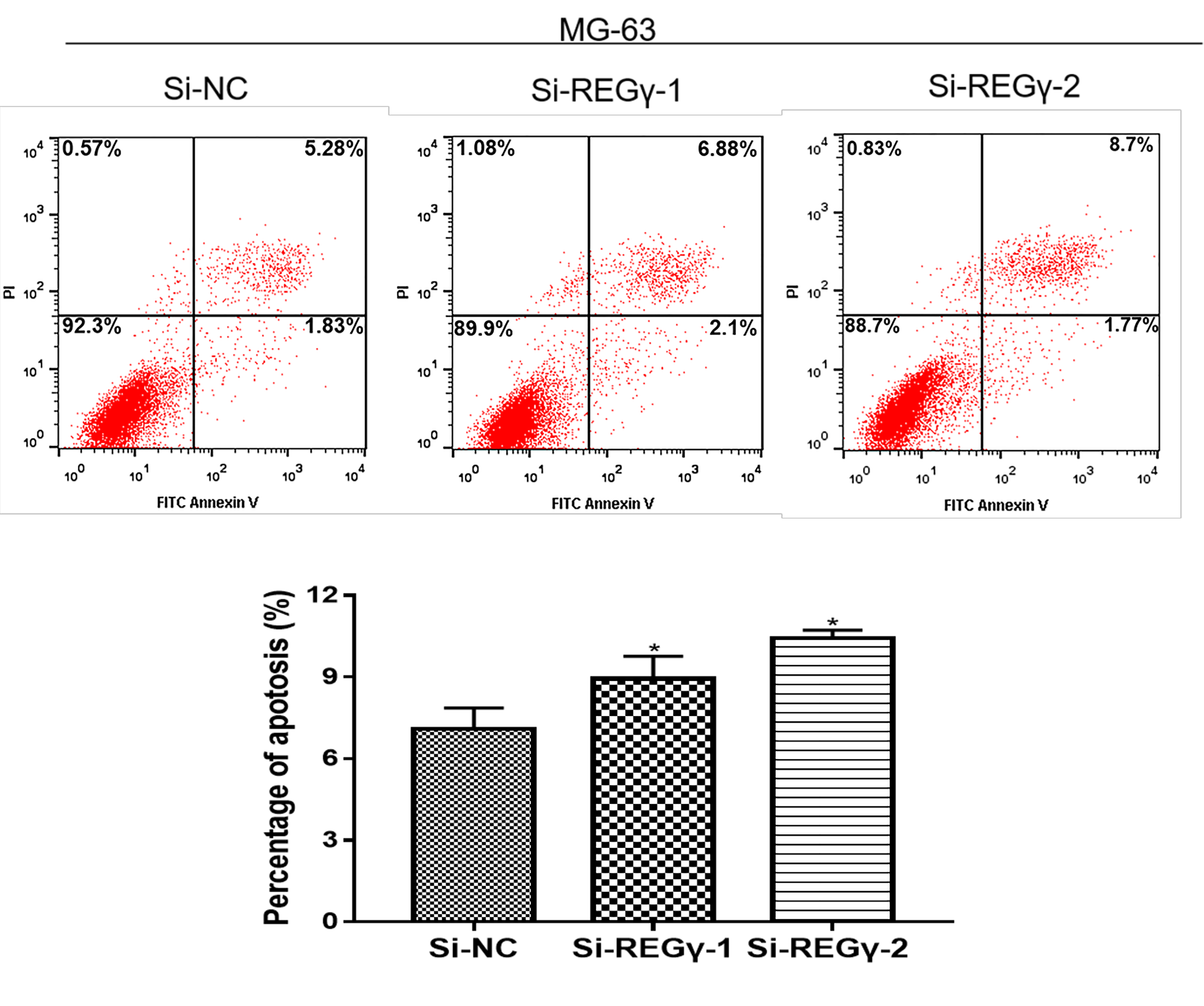

Supplement: Supplemental Information 6 [file peerj-08-8954-s006.zip › Figure 4 raw data-1/FCM-apoptosis/mg-63/mg-63 apotosis-tiff,600dpi.tif]

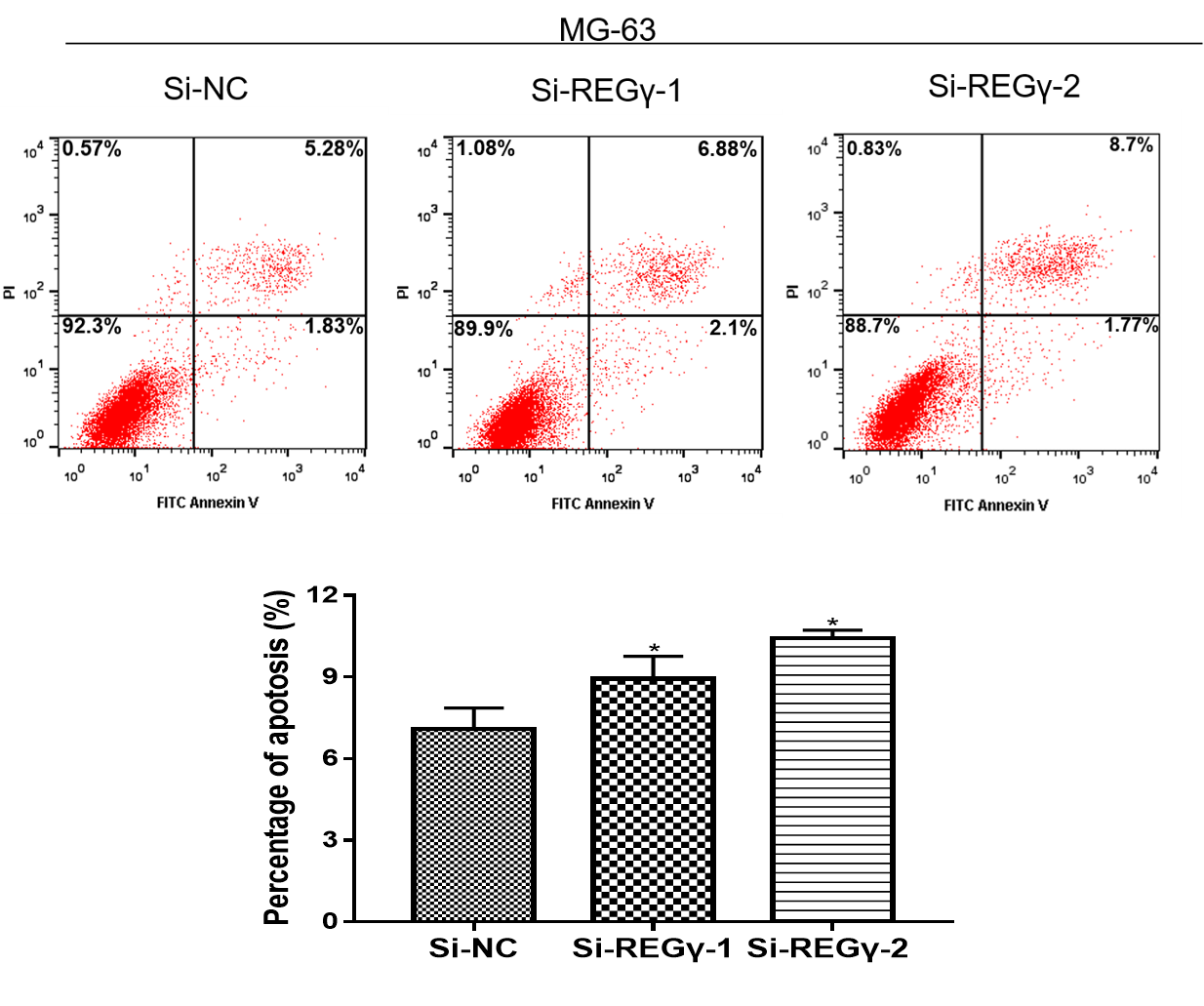

Supplement: Supplemental Information 6 [file peerj-08-8954-s006.zip › Figure 4 raw data-1/FCM-apoptosis/mg-63/mg-63 apotosis.png]

## Slide 1
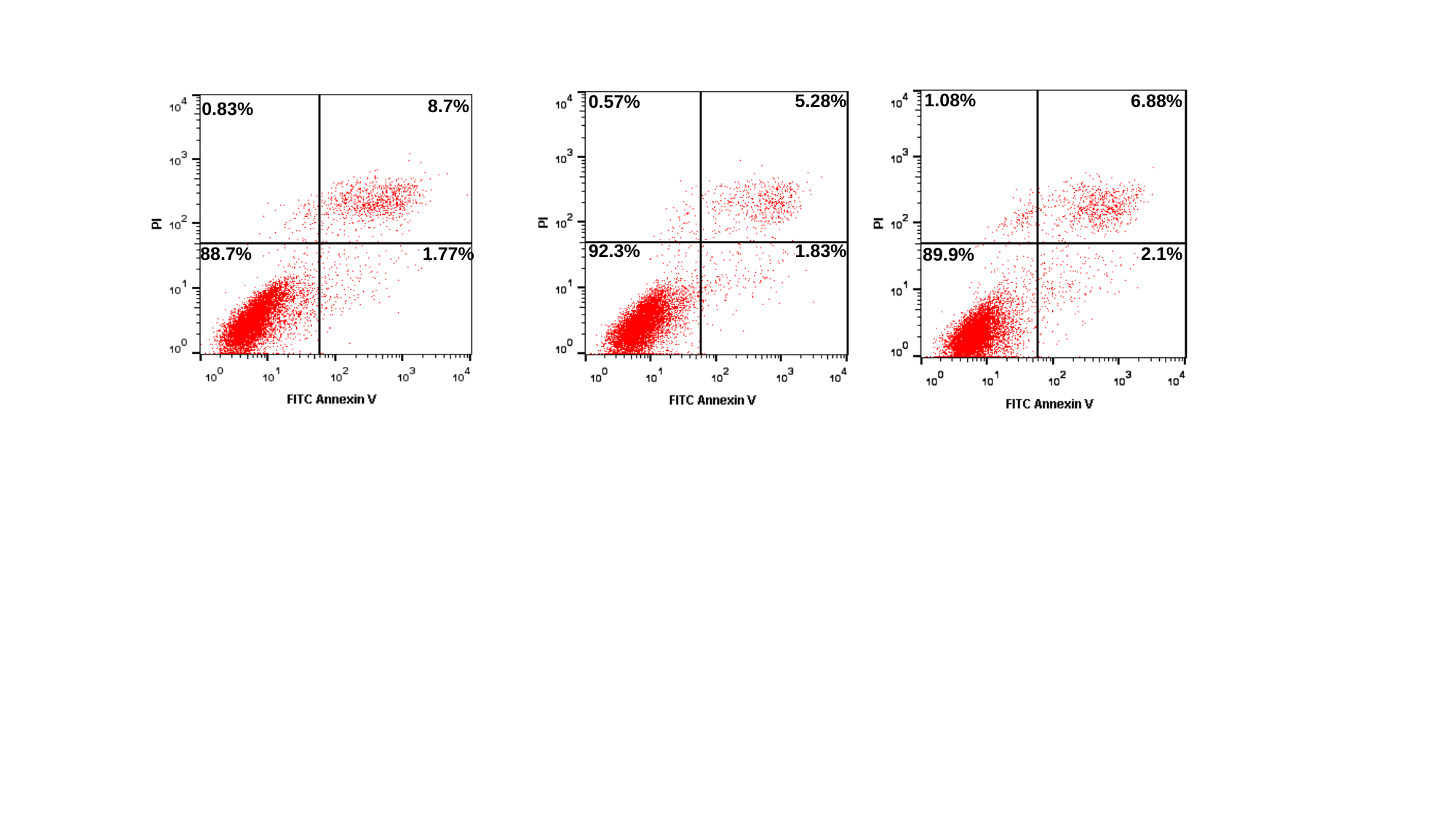

5.28%
0.57%
92.3%
1.83%
1.08%
6.88%
2.1%
89.9%
8.7%
0.83%
1.77%
88.7%

## Slide 2
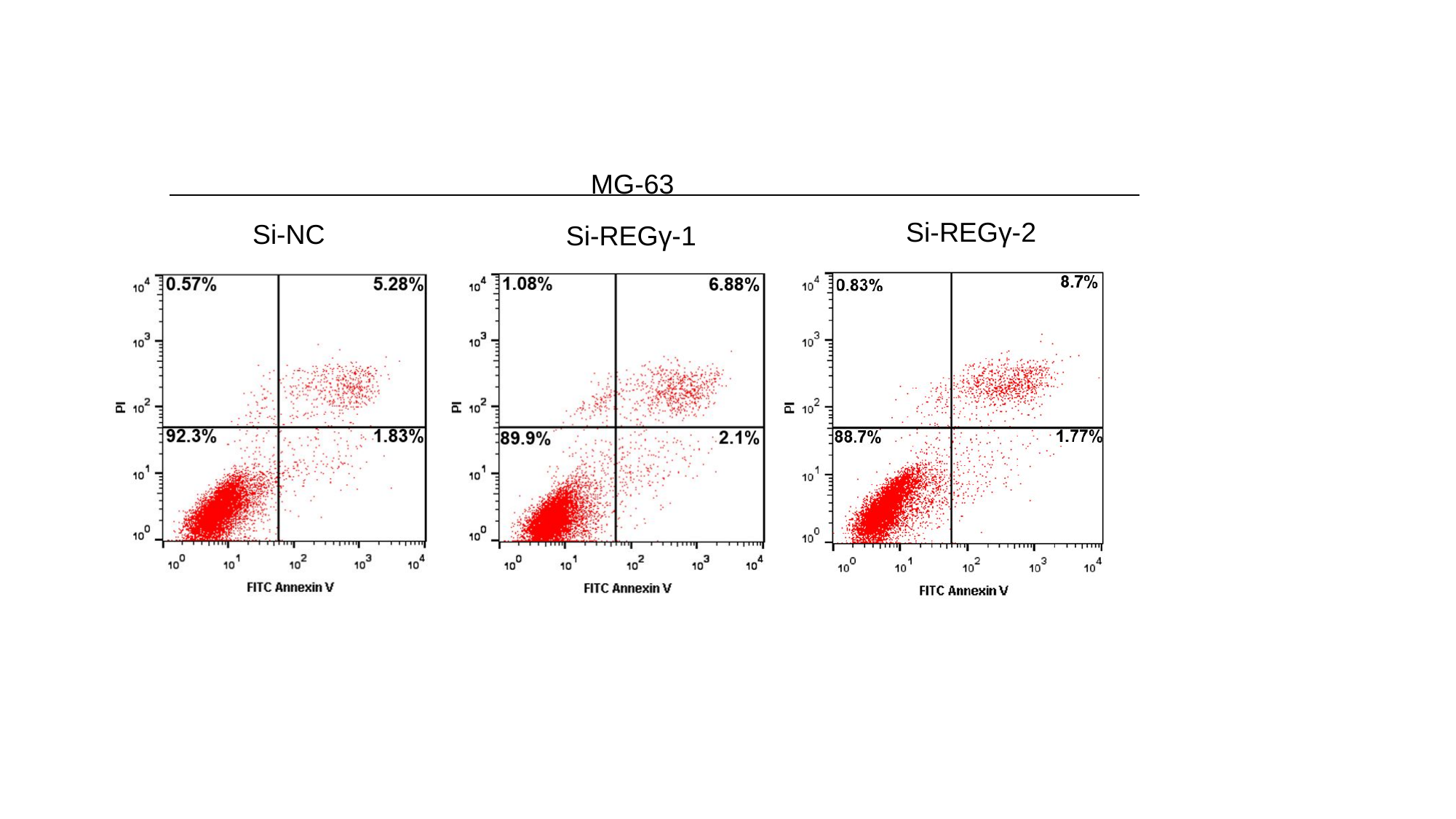

MG-63
Si-REGγ-2
Si-NC
 Si-REGγ-1

## Slide 3
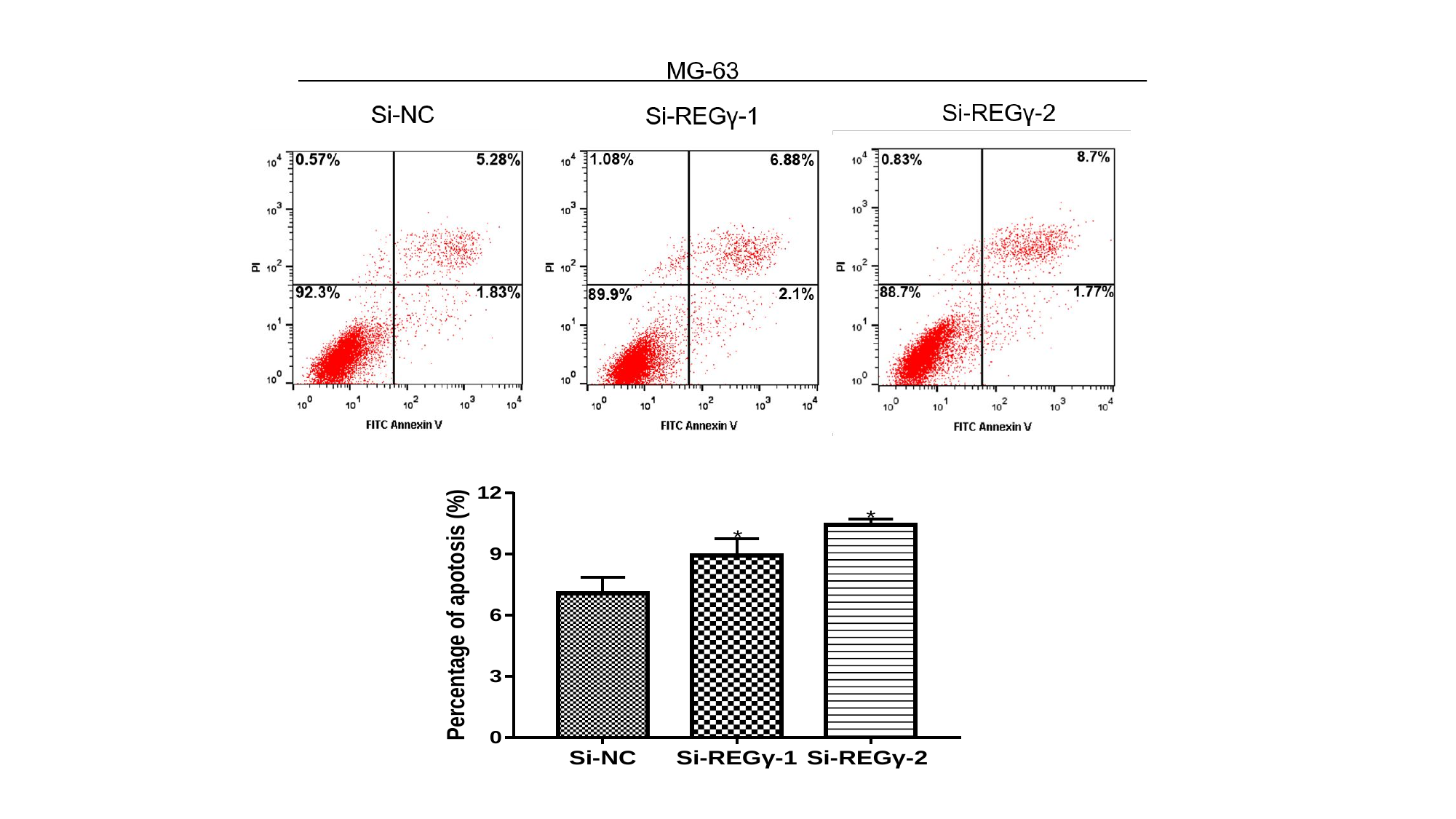

Supplement: Supplemental Information 6 [file peerj-08-8954-s006.zip › Figure 4 raw data-1/FCM-apoptosis/mg-63/做图留存.pptx]

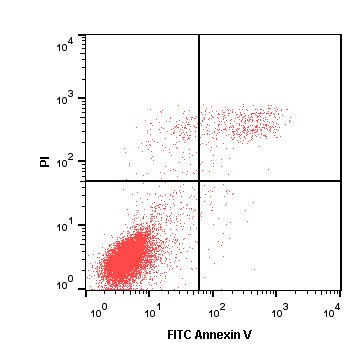

Supplement: Supplemental Information 6 [file peerj-08-8954-s006.zip › Figure 4 raw data-1/FCM-apoptosis/SaoS-2/1-Layout.png]

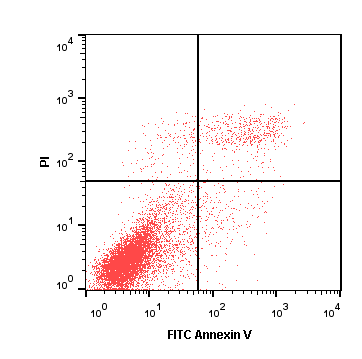

Supplement: Supplemental Information 6 [file peerj-08-8954-s006.zip › Figure 4 raw data-1/FCM-apoptosis/SaoS-2/2-Layout.png]

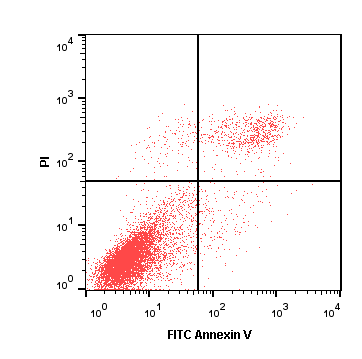

Supplement: Supplemental Information 6 [file peerj-08-8954-s006.zip › Figure 4 raw data-1/FCM-apoptosis/SaoS-2/3-Layout.png]

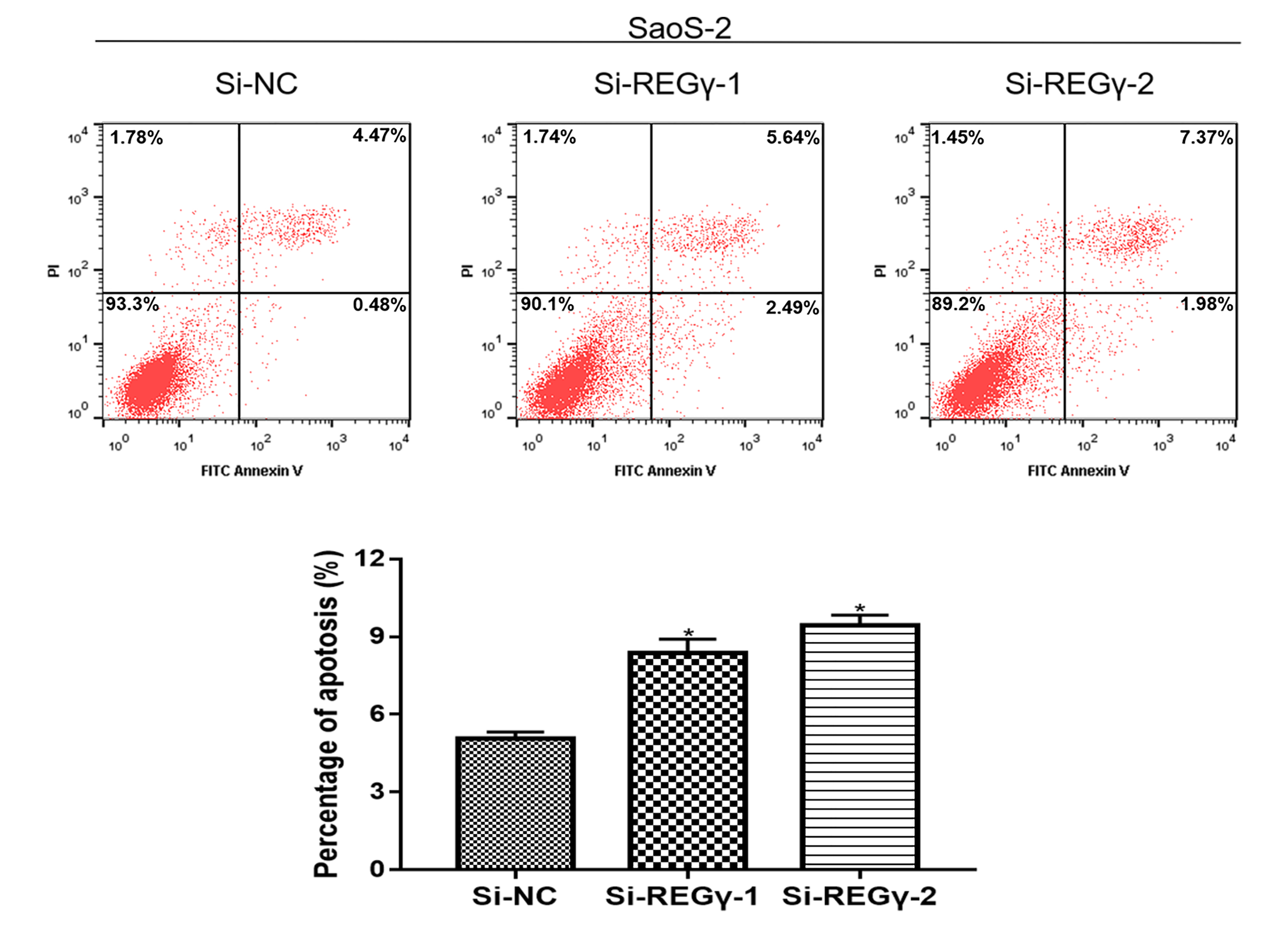

Supplement: Supplemental Information 6 [file peerj-08-8954-s006.zip › Figure 4 raw data-1/FCM-apoptosis/SaoS-2/saoS-2 apoptosis-tiff-600dpi.tif]

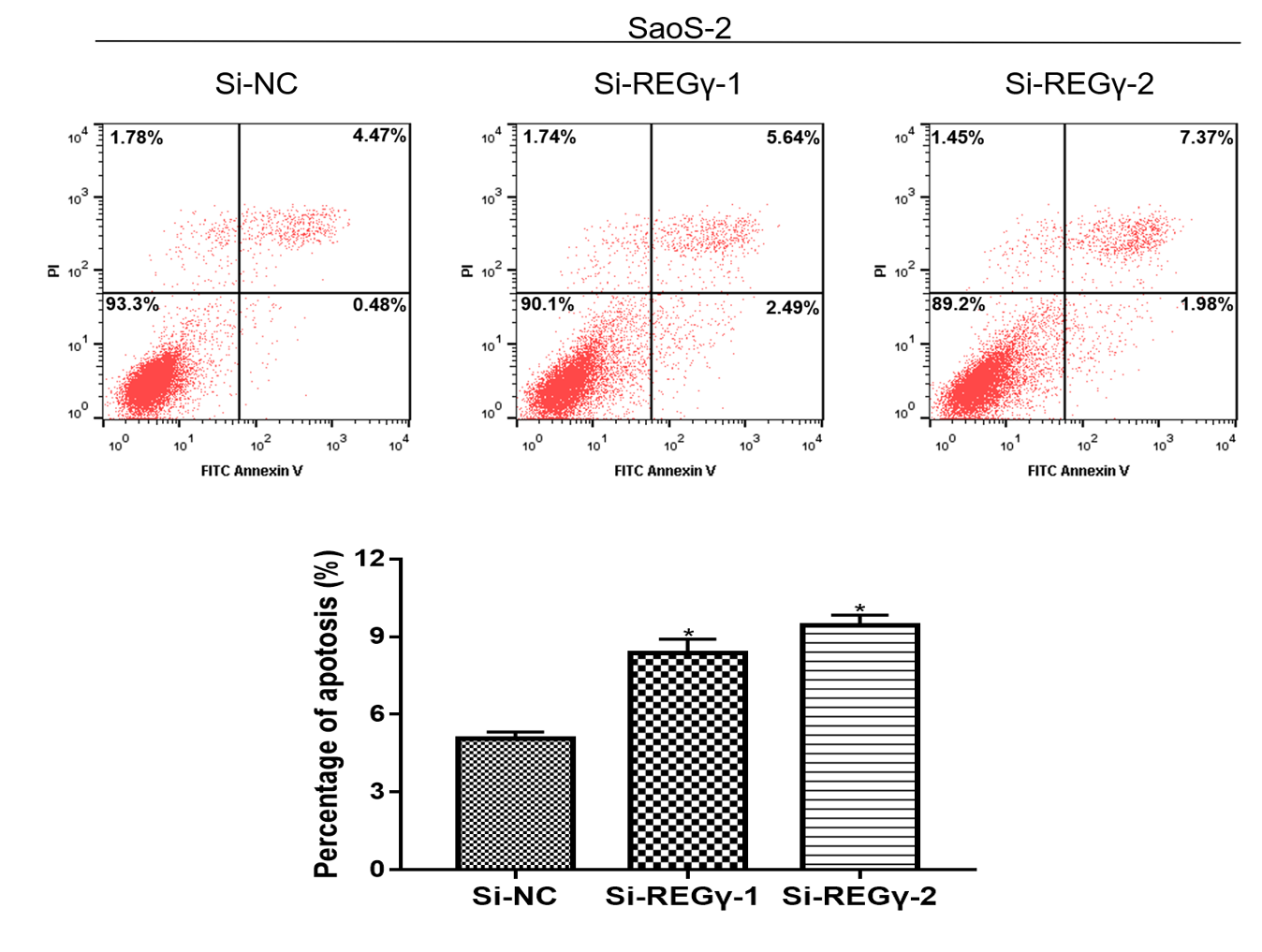

Supplement: Supplemental Information 6 [file peerj-08-8954-s006.zip › Figure 4 raw data-1/FCM-apoptosis/SaoS-2/saoS-2 apoptosis.png]

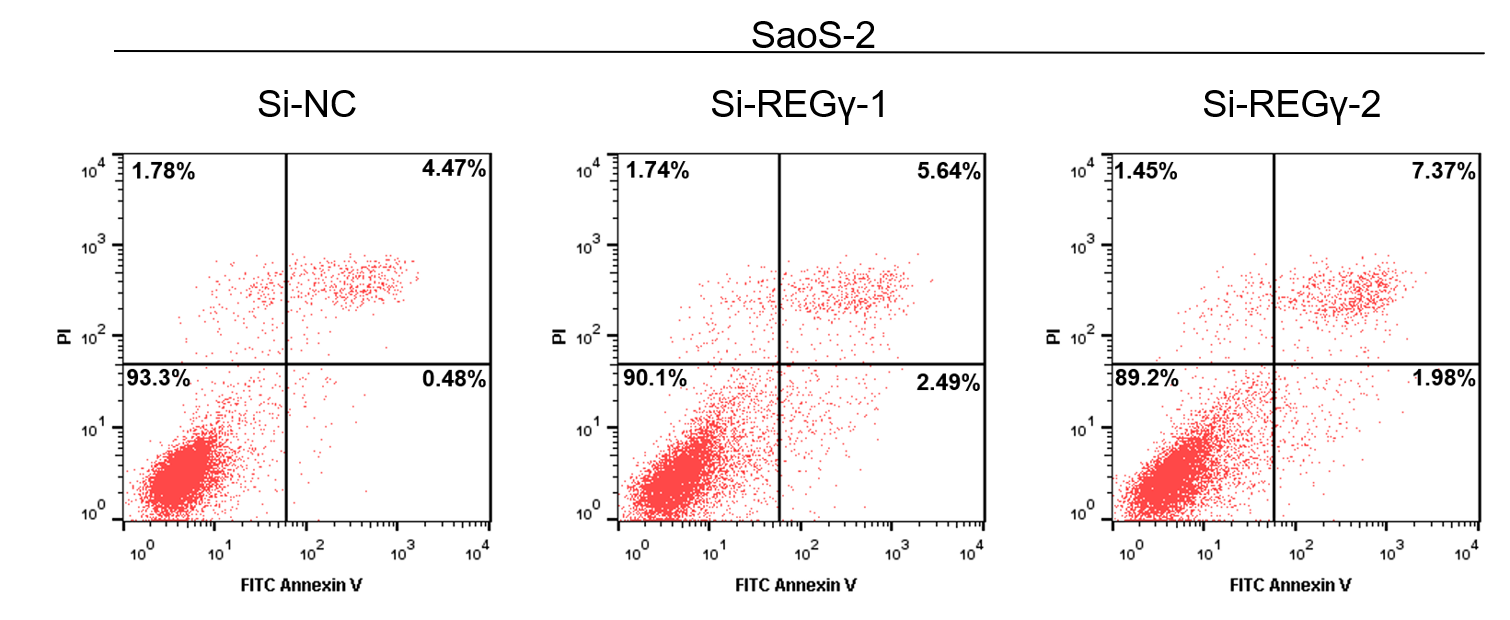

Supplement: Supplemental Information 6 [file peerj-08-8954-s006.zip › Figure 4 raw data-1/FCM-apoptosis/SaoS-2/zuhe.png]

## Slide 1
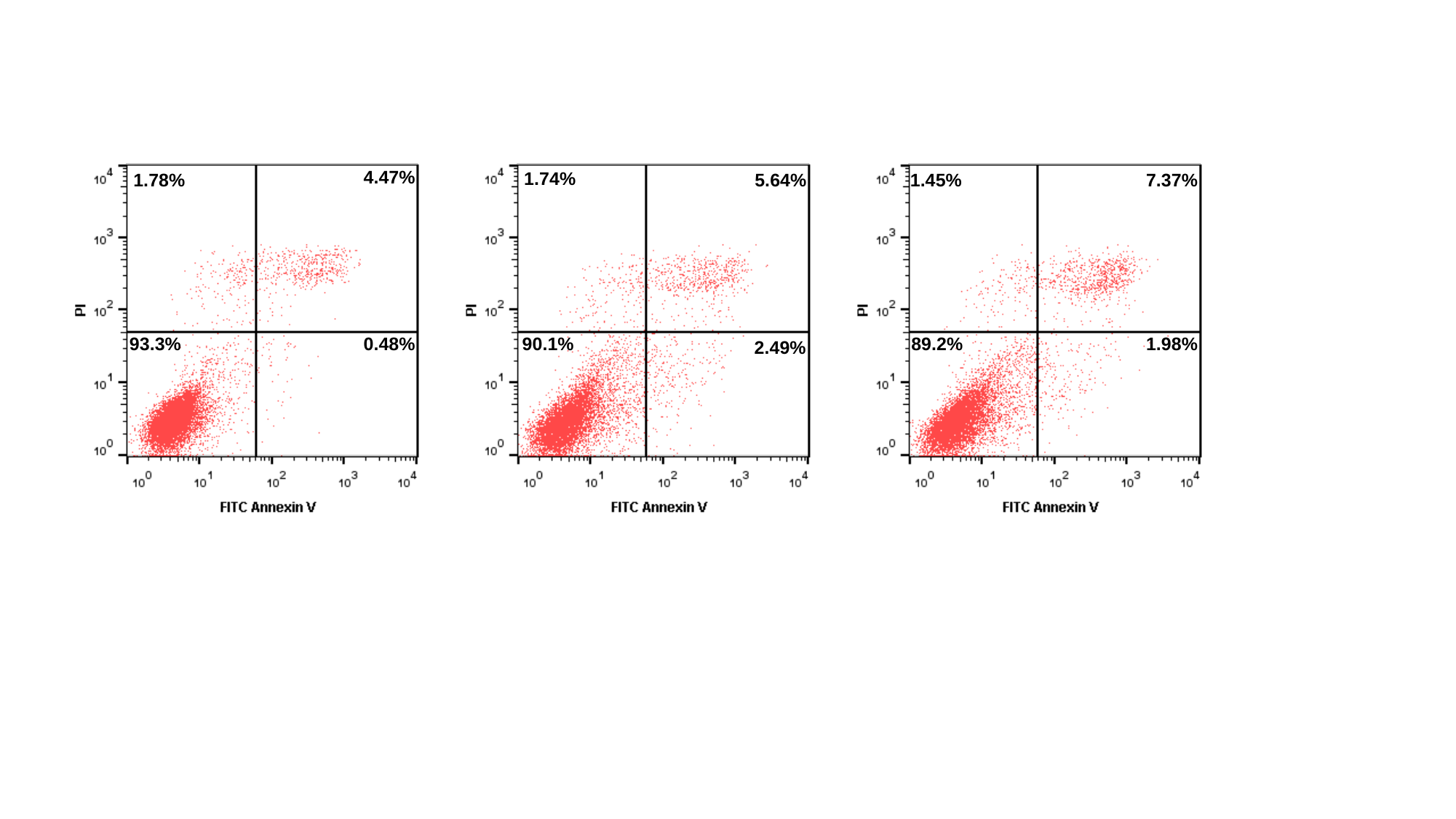

4.47%
1.74%
1.45%
7.37%
5.64%
1.78%
89.2%
1.98%
90.1%
93.3%
0.48%
2.49%

## Slide 2
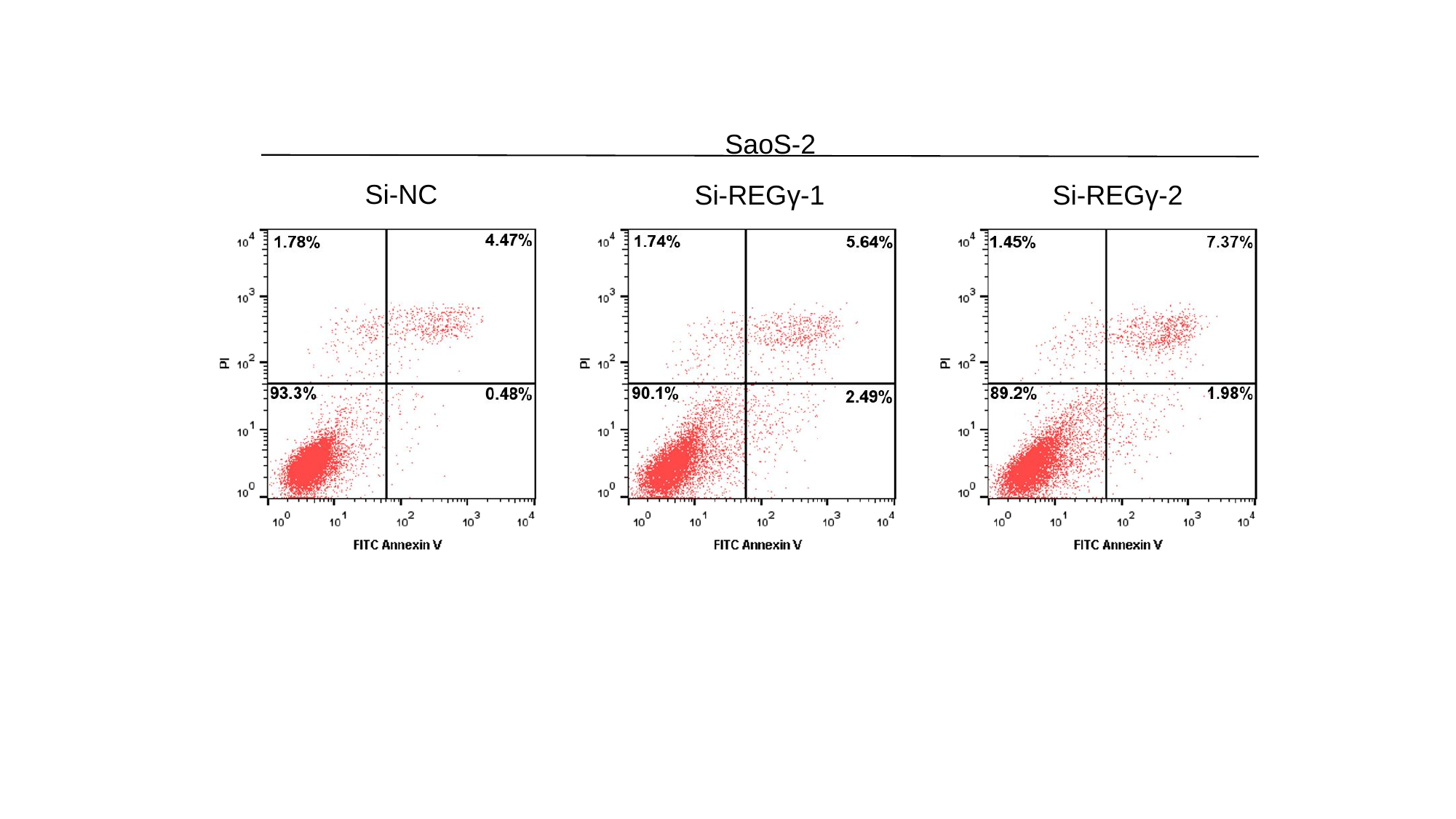

SaoS-2
Si-NC
 Si-REGγ-1
 Si-REGγ-2

## Slide 3
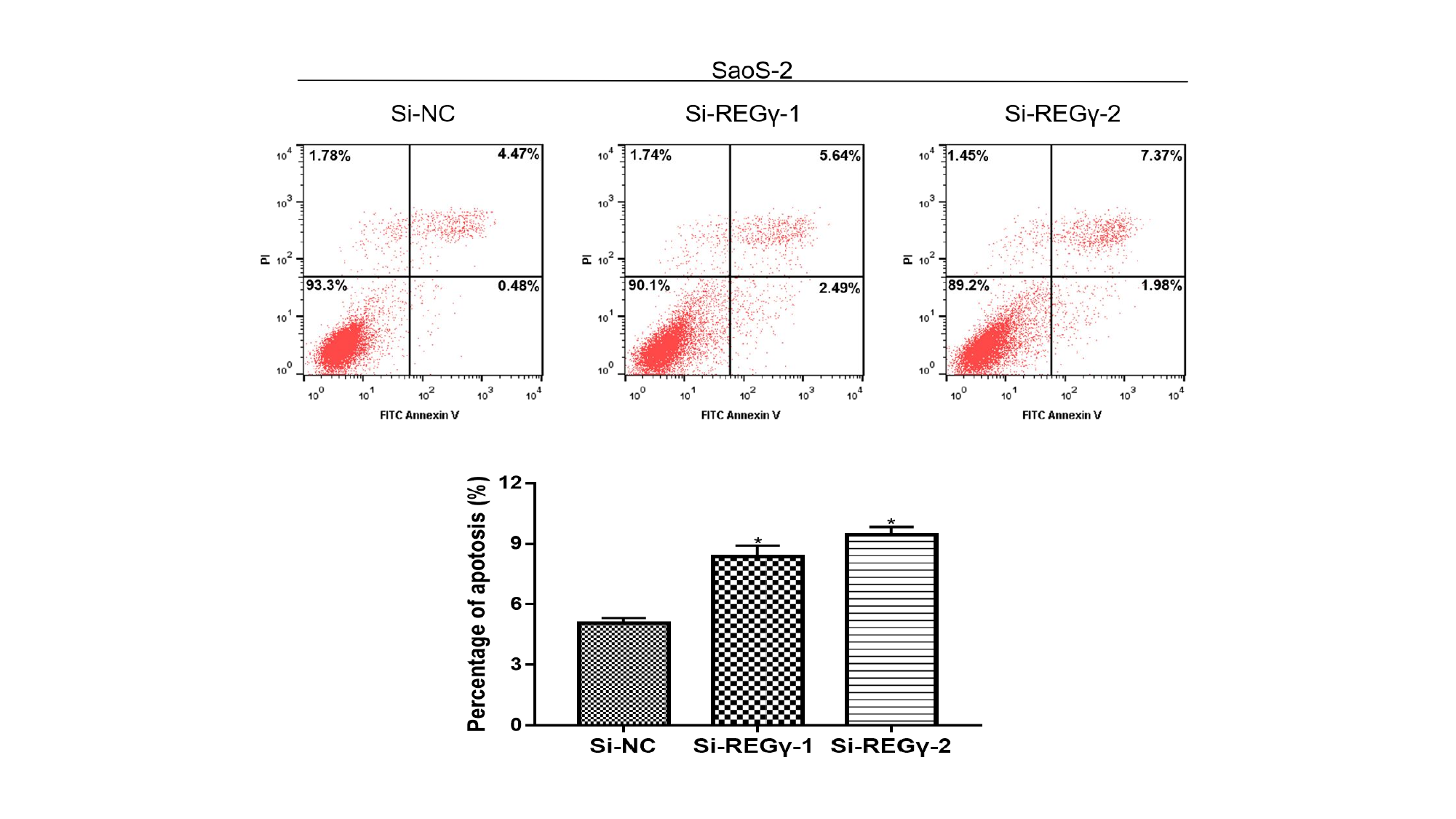

## Slide 4
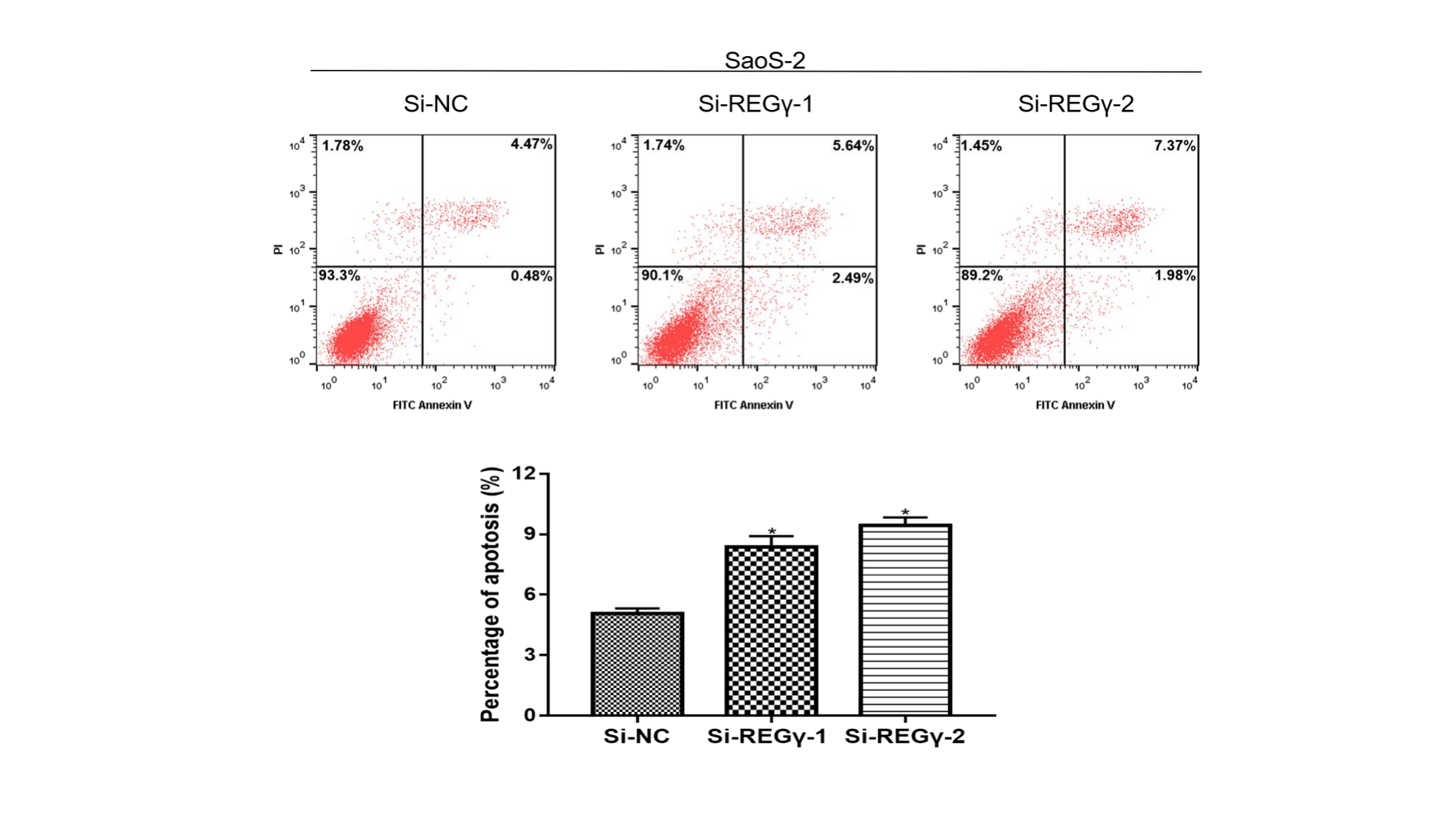

Supplement: Supplemental Information 6 [file peerj-08-8954-s006.zip › Figure 4 raw data-1/FCM-apoptosis/SaoS-2/做图留存.pptx]

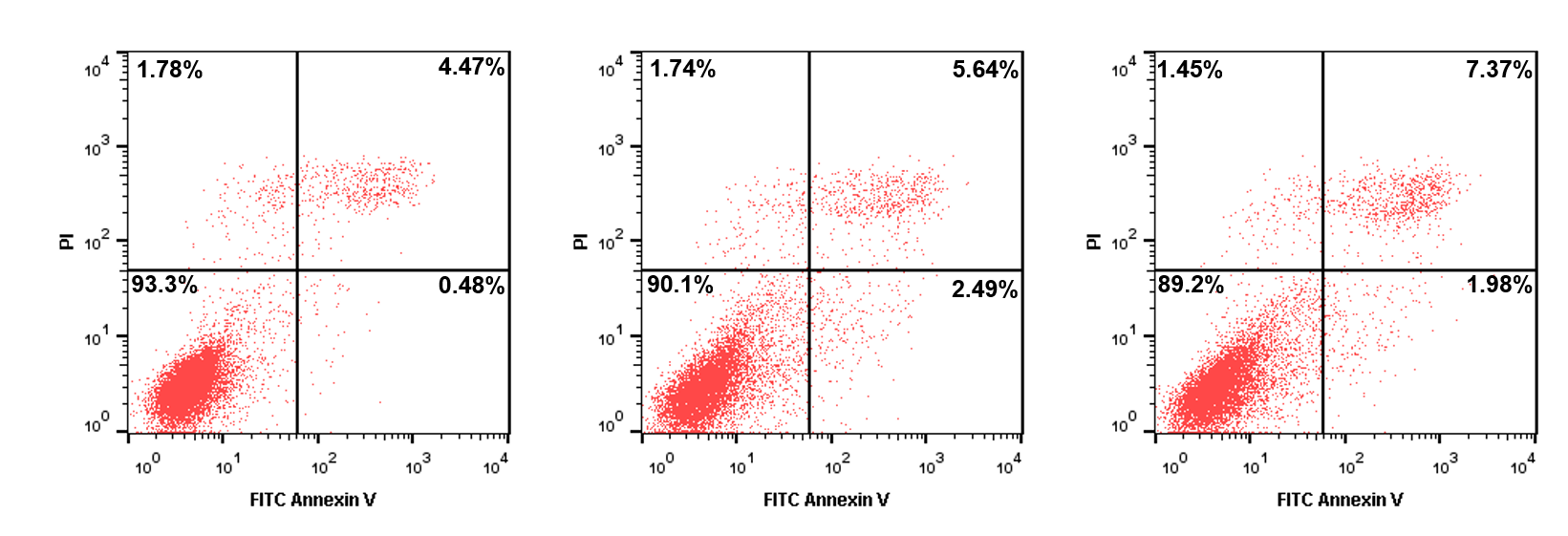

Supplement: Supplemental Information 6 [file peerj-08-8954-s006.zip › Figure 4 raw data-1/FCM-apoptosis/SaoS-2/图片1.png]

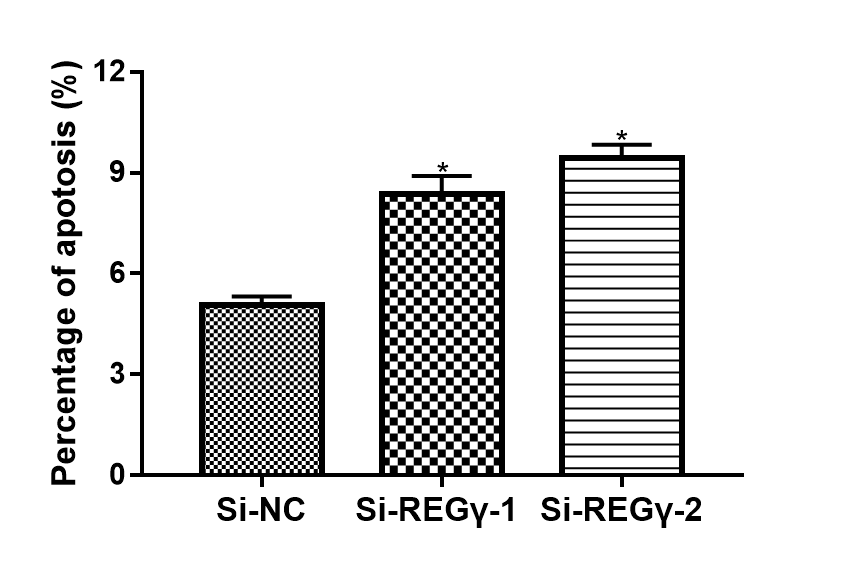

Supplement: Supplemental Information 6 [file peerj-08-8954-s006.zip › Figure 4 raw data-1/FCM-apoptosis/SaoS-2/图片统计.png]

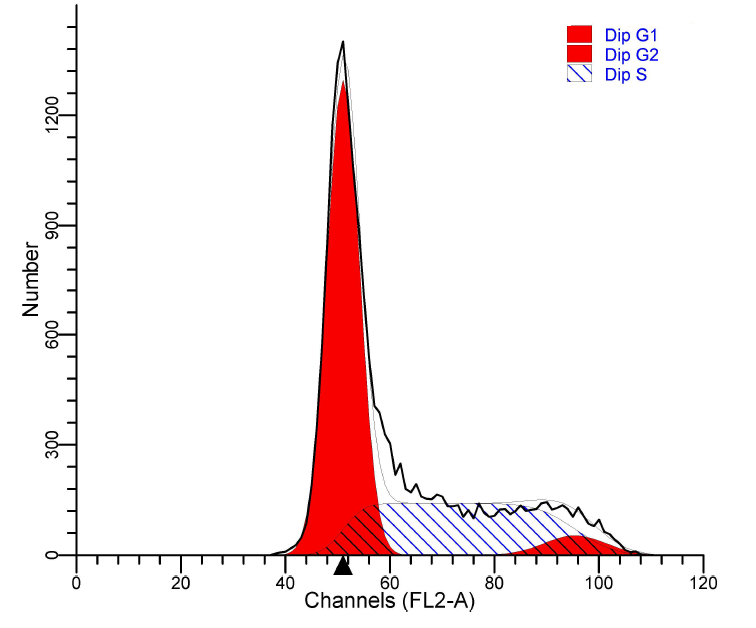

Supplement: Supplemental Information 6 [file peerj-08-8954-s006.zip › Figure 4 raw data-1/FCM-cell cycle/MG-63/1.png]

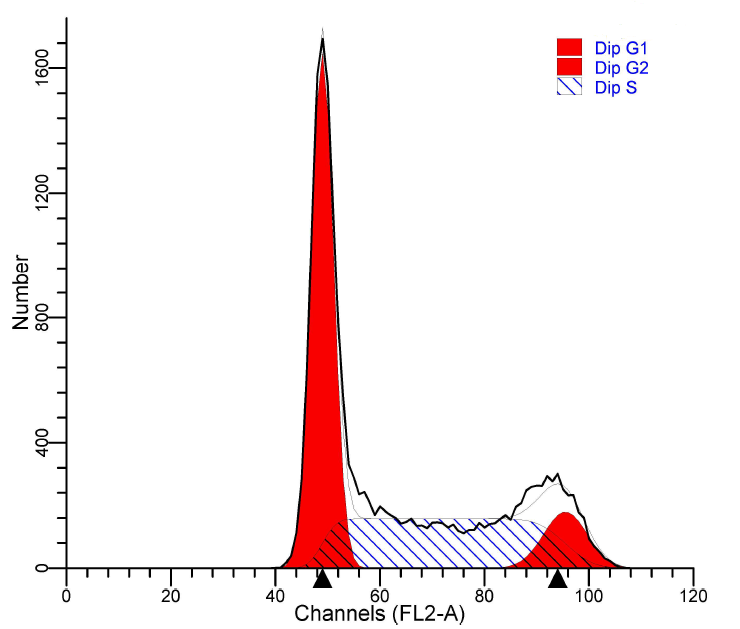

Supplement: Supplemental Information 6 [file peerj-08-8954-s006.zip › Figure 4 raw data-1/FCM-cell cycle/MG-63/2.png]

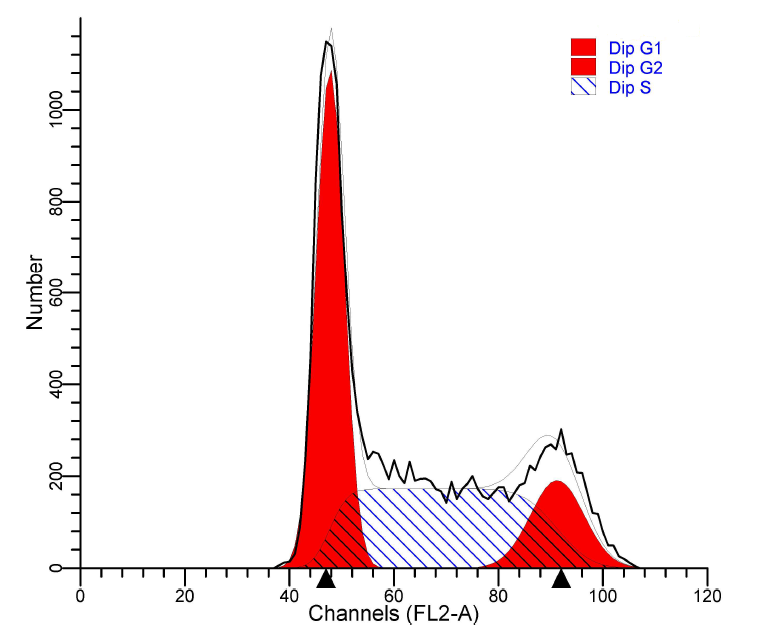

Supplement: Supplemental Information 6 [file peerj-08-8954-s006.zip › Figure 4 raw data-1/FCM-cell cycle/MG-63/3.png]

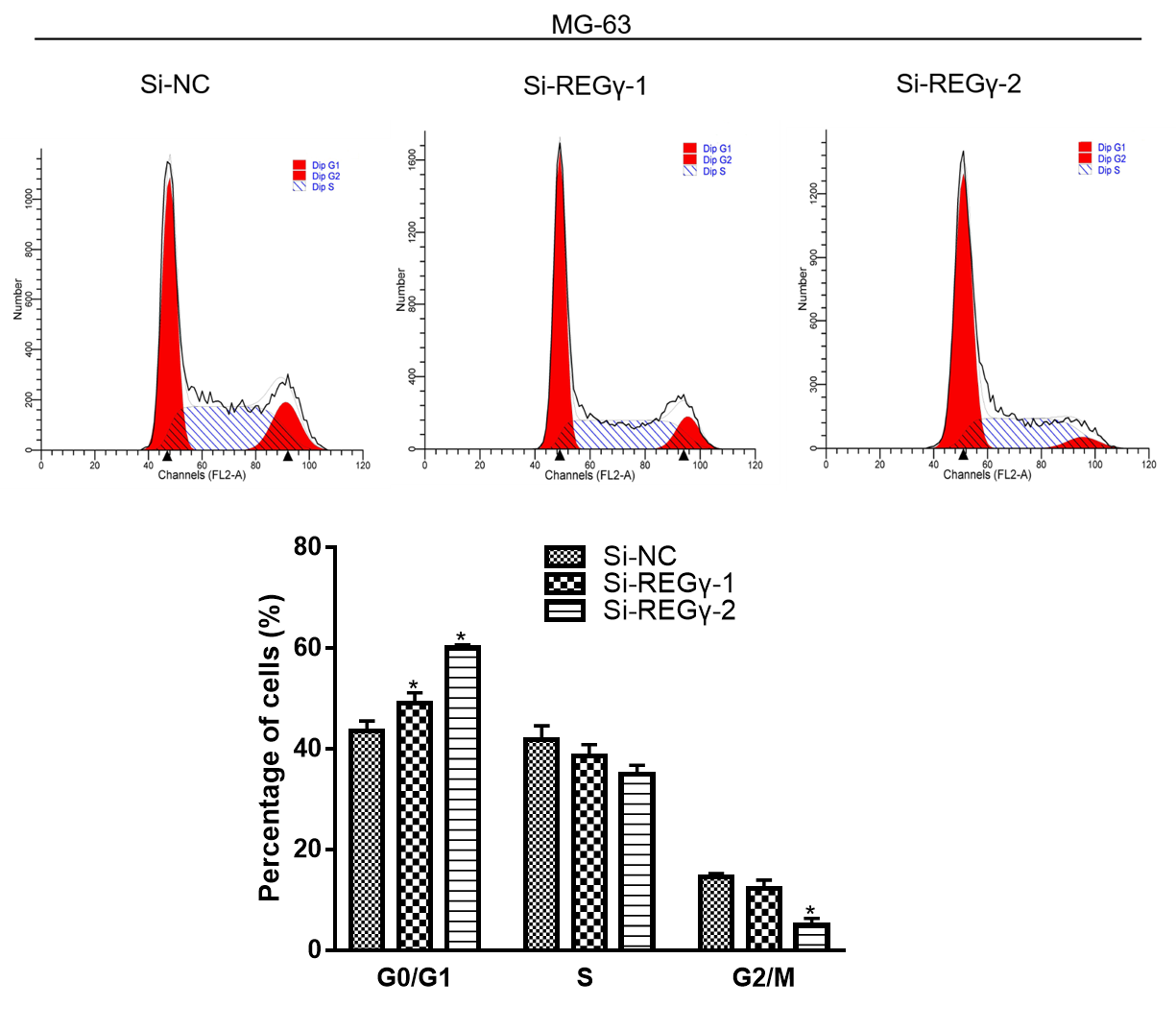

Supplement: Supplemental Information 6 [file peerj-08-8954-s006.zip › Figure 4 raw data-1/FCM-cell cycle/MG-63/mg-63 cell cycle complete.png]

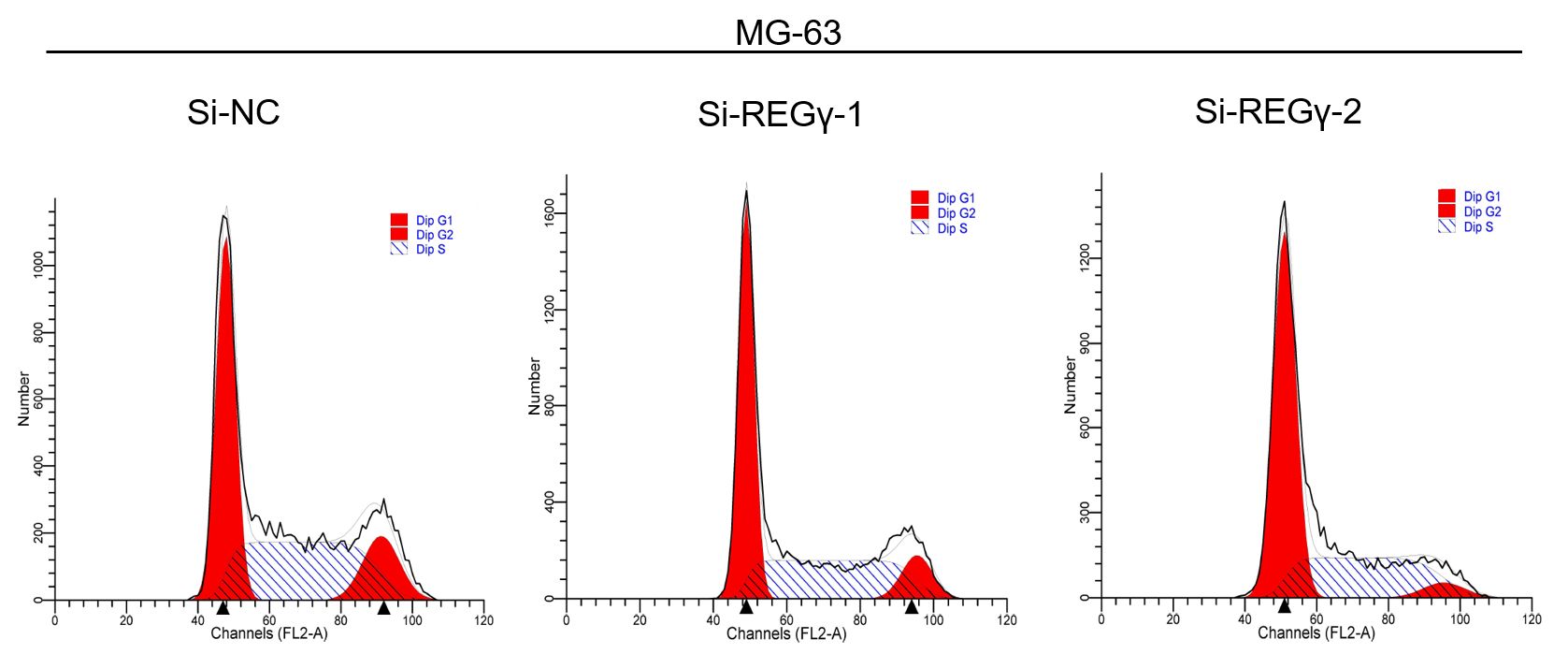

Supplement: Supplemental Information 6 [file peerj-08-8954-s006.zip › Figure 4 raw data-1/FCM-cell cycle/MG-63/MG-63 cell cycle.png]

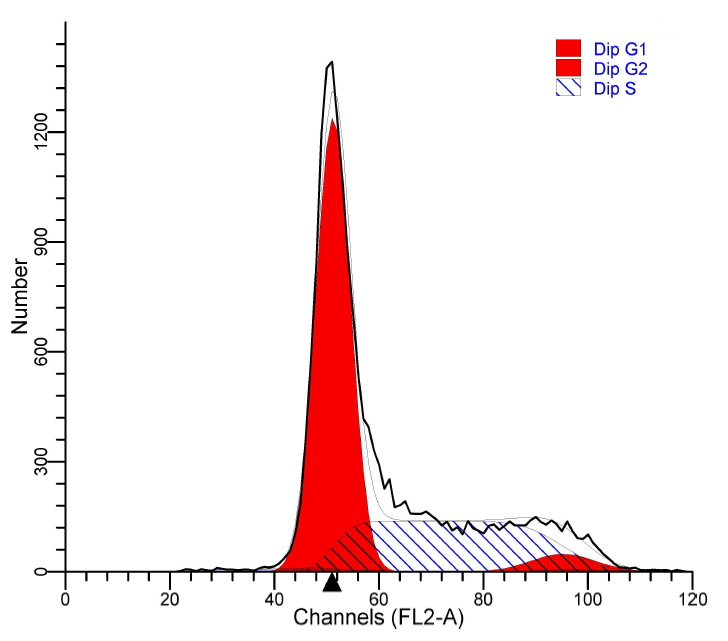

Supplement: Supplemental Information 6 [file peerj-08-8954-s006.zip › Figure 4 raw data-1/FCM-cell cycle/Saos-2/1.png]

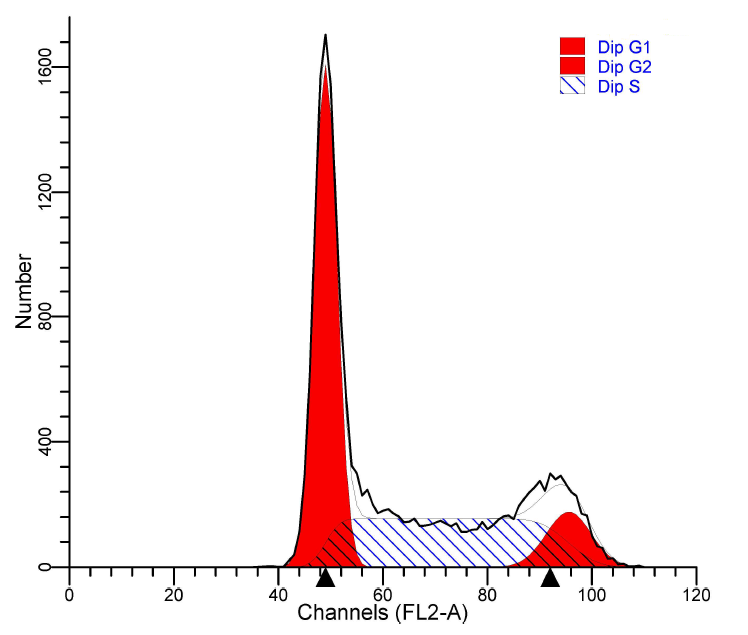

Supplement: Supplemental Information 6 [file peerj-08-8954-s006.zip › Figure 4 raw data-1/FCM-cell cycle/Saos-2/2.png]

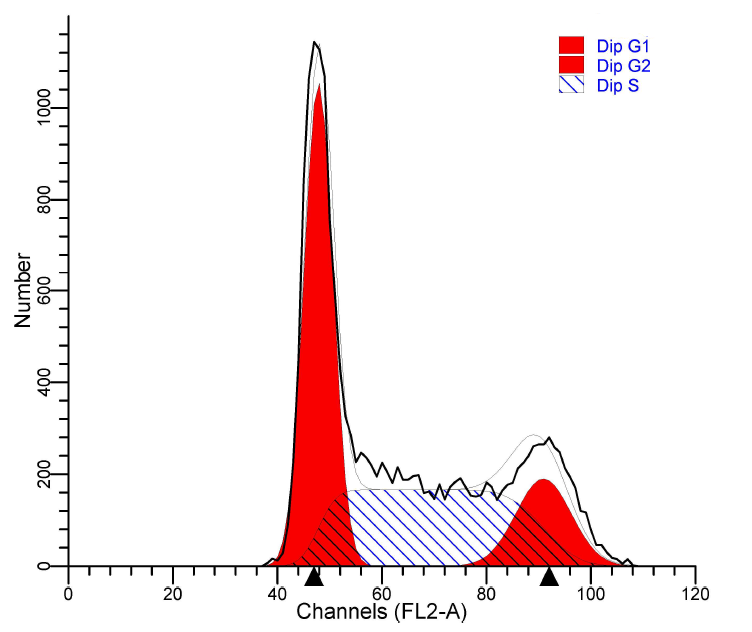

Supplement: Supplemental Information 6 [file peerj-08-8954-s006.zip › Figure 4 raw data-1/FCM-cell cycle/Saos-2/3.png]

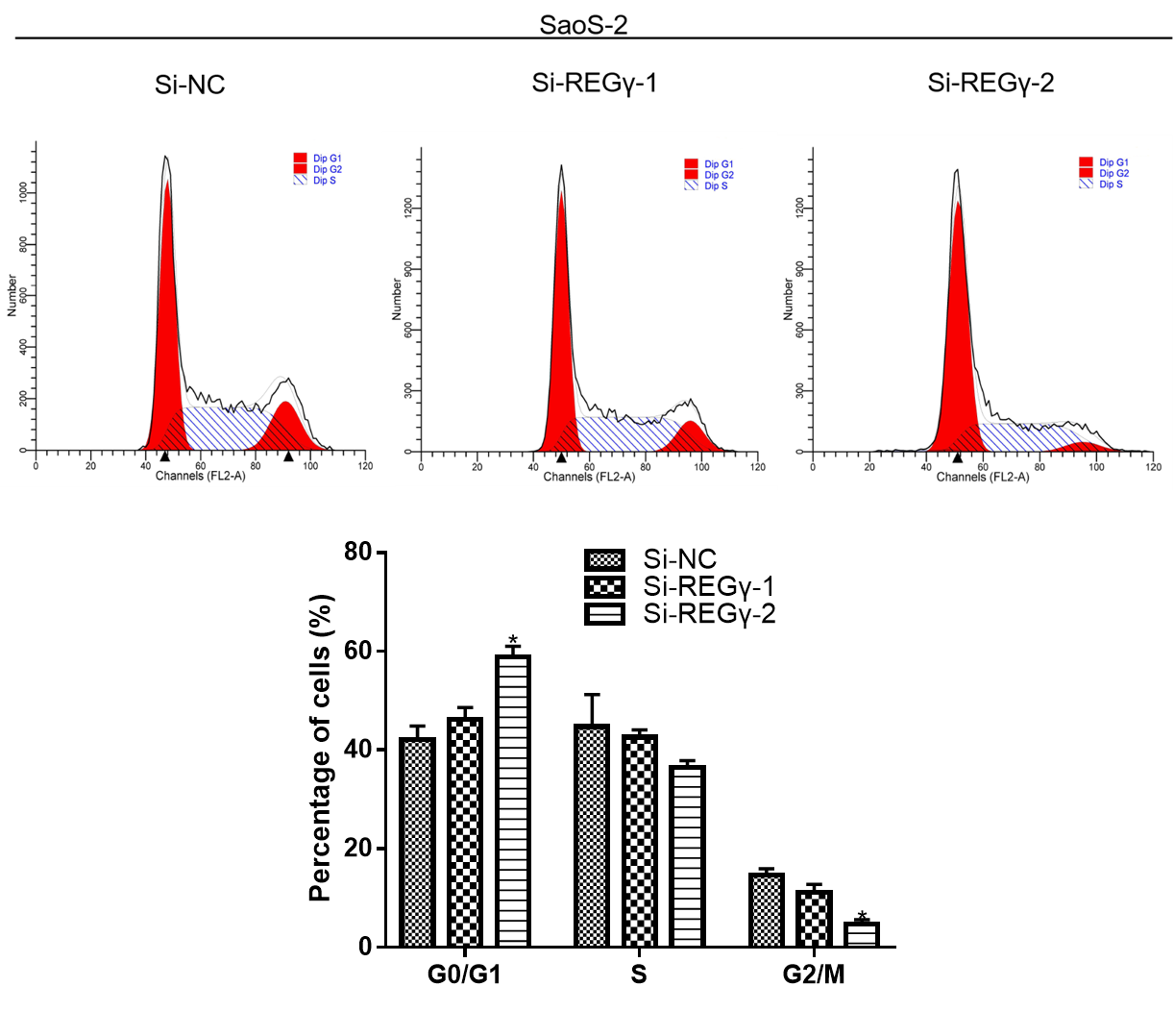

Supplement: Supplemental Information 6 [file peerj-08-8954-s006.zip › Figure 4 raw data-1/FCM-cell cycle/Saos-2/SaoS-2 cell cycle complete.png]

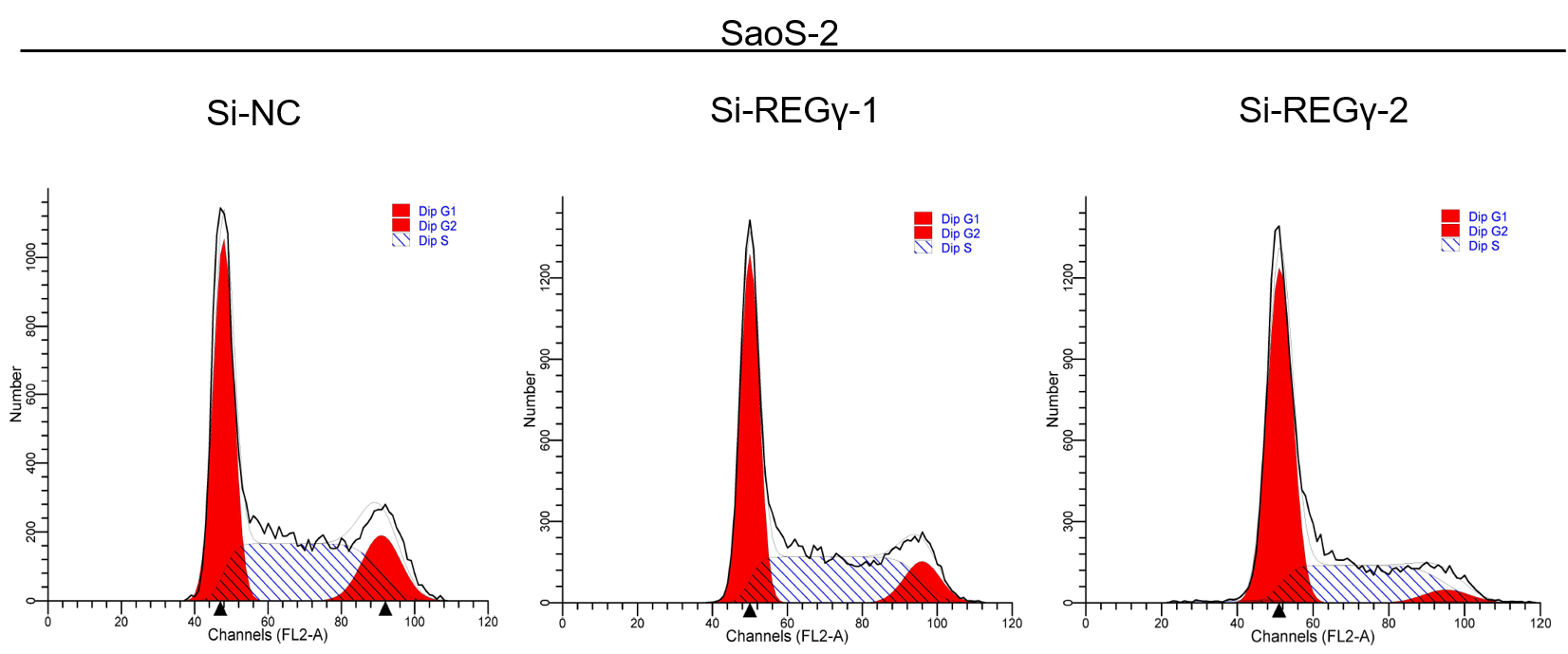

Supplement: Supplemental Information 6 [file peerj-08-8954-s006.zip › Figure 4 raw data-1/FCM-cell cycle/Saos-2/SaoS-2.png]

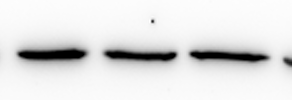

Supplement: Supplemental Information 7 [file peerj-08-8954-s007.zip › Figure 4 raw data-2/actin mg63.png]

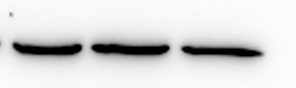

Supplement: Supplemental Information 7 [file peerj-08-8954-s007.zip › Figure 4 raw data-2/actin saos2.png]

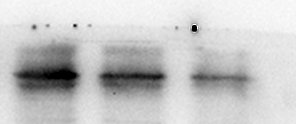

Supplement: Supplemental Information 7 [file peerj-08-8954-s007.zip › Figure 4 raw data-2/bcl-2 mg63.png]

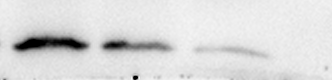

Supplement: Supplemental Information 7 [file peerj-08-8954-s007.zip › Figure 4 raw data-2/bcl-2 saos2.png]

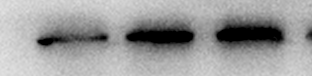

Supplement: Supplemental Information 7 [file peerj-08-8954-s007.zip › Figure 4 raw data-2/caspase-3 mg63 .png]

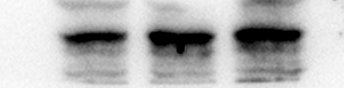

Supplement: Supplemental Information 7 [file peerj-08-8954-s007.zip › Figure 4 raw data-2/caspase-3 saos2.png]

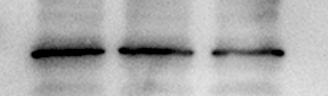

Supplement: Supplemental Information 7 [file peerj-08-8954-s007.zip › Figure 4 raw data-2/cyclinD mg63.png]

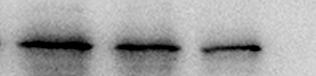

Supplement: Supplemental Information 7 [file peerj-08-8954-s007.zip › Figure 4 raw data-2/cyclinD saos2.png]

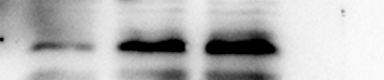

Supplement: Supplemental Information 7 [file peerj-08-8954-s007.zip › Figure 4 raw data-2/p21 mg63.png]

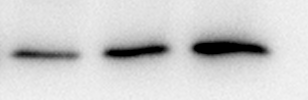

Supplement: Supplemental Information 7 [file peerj-08-8954-s007.zip › Figure 4 raw data-2/p21 saos2.png]

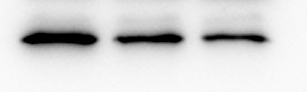

Supplement: Supplemental Information 7 [file peerj-08-8954-s007.zip › Figure 4 raw data-2/REGγ saos2.png]

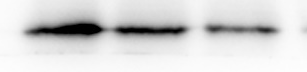

Supplement: Supplemental Information 7 [file peerj-08-8954-s007.zip › Figure 4 raw data-2/REGγ mg63.png]
